# RNA‐Protein Assemblies: A Review of Biophysical Principles and Coarse‐Grained Modeling Approaches

**DOI:** 10.1002/wrna.70061

**Published:** 2026-10-02

**Authors:** Muhammad Waleed Iqbal, Patrick R. Onck

**Affiliations:** ^1^ Zernike Institute for Advanced Materials University of Groningen Groningen the Netherlands

**Keywords:** nuclear speckles, nucleoli, RNA‐protein assemblies, vRNP

## Abstract

RNA‐protein assemblies are fundamental organizers of eukaryotic cell biology, participating in diverse processes such as ribosome biogenesis in nucleoli, mRNA sequestration in nuclear speckles, and genome packaging in viral ribonucleoprotein (vRNP) complexes. Across these systems, multivalent RNA‐protein interactions promote self‐organization, contributing directly to liquid–liquid phase separation (LLPS) in nucleoli and nuclear speckles, while facilitating assembly and intermediate states in mature structurally organized vRNPs. This review critically examines the molecular grammar underlying these three paradigmatic systems, focusing on how sequence‐encoded RNA‐IDR chemistry determines condensate morphology, compositional selectivity, and material properties. We then evaluate the experimental and computational approaches used to characterize RNA‐protein assemblies, with particular focus on coarse‐grained (CG) forcefield development across HPS, Mpipi, CALVADOS, and 1BPA families. We also discuss how emerging multi‐domain protein (MDP) models and RNA‐compatible forcefields are optimizing the predictive accuracy of molecular simulations. Finally, we explore physics‐based and data‐driven predictors of LLPS, and reiterate the need for a framework that can predict sequence‐based heterotypic IDR‐RNA co‐phase separation.

## Graphical abstract

RNA‐protein assemblies in the nucleolus, nuclear speckles, and viral ribonucleoprotein complexes are governed by multivalent IDR‐RNA interactions that drive liquid–liquid phase separation. This review examines the molecular grammar underlying these systems and critically evaluates coarse‐grained computational frameworks for modeling their sequence‐dependent condensate formation and material properties.

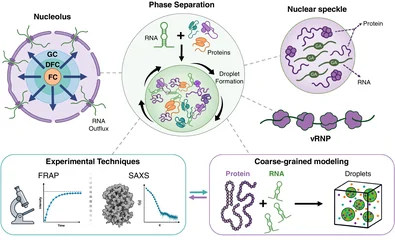

## 1 Introduction

Biomolecular assemblies are crucial for cellular organization, acting to facilitate ribosome biogenesis, stress response, viral replication, and regulation of gene expression (Banani et al. 2017; Lyon et al. 2021). These assemblies include phase‐separated condensates and structurally stable complexes, which bring biomolecules together in dynamic and biophysically distinct microenvironments (Shin and Brangwynne 2017). The structure and function of membraneless compartments (such as the nucleolus) and stable complexes (such as viral protein assemblies) are critically dependent on the molecular mechanism of self‐organization resulting from multivalent RNA‐protein interactions (Iserman et al. 2020; Lafontaine et al. 2021). A major distinction of biomolecular condensates from classical membrane‐bound compartments is their ability to undergo liquid–liquid phase separation (LLPS) driven by macromolecular interactions, which allow for the spontaneous segregation of molecules into dense and dilute phases (Brangwynne et al. 2009; Hyman et al. 2014). For example, the nucleolus undergoes hierarchical phase separation into three subcompartments via biophysical macromolecular interactions, which result in the formation of an innermost fibrillar center (FC), surrounded by a spherical dense fibrillar component (DFC), and an outer granular component (GC) which encapsulates the DFC (see Figure 1A) (Feric et al. 2016; Lafontaine et al. 2021). This inter‐compartmental immiscibility is required to assemble and process ribosomal subunits through RNA‐protein multivalent interactions in a series of coordinated sequential steps (Yao et al. 2019). Equally important, nuclear speckles represent a distinct class of membraneless organelles (MLOs) enriched in pre‐mRNA splicing factors and RNA‐binding proteins (RBPs), where sequence‐specific mRNA‐protein interactions regulate mRNA sequestration and export, maintaining cellular homeostasis (see Figure 1B) (Faraway et al. 2025; Galganski et al. 2017). Beyond nuclear bodies, growing evidence suggests that RNA‐protein multivalent interactions in viruses, such as the SARS‐CoV‐2 ribonucleoprotein complexes, may also lead to distinct viral complexes through LLPS, playing a key role in genome packaging, RNA replication, and assembly (Carlson et al. 2022; Savastano et al. 2020) (see Figure 1C).

**FIGURE 1:**
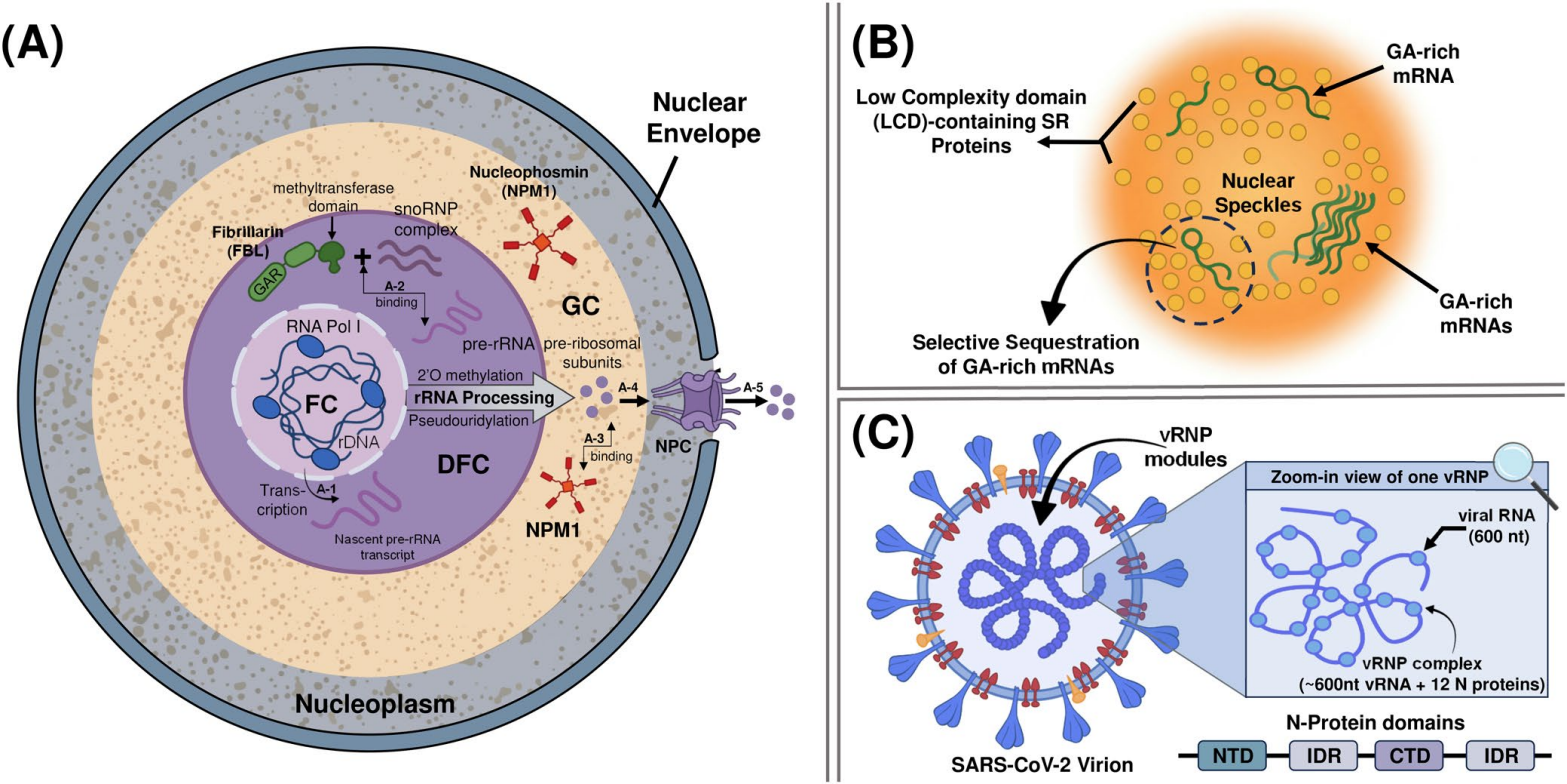
Schematic representation of three paradigmatic RNA‐protein assemblies. (A) The nucleolus undergoes hierarchical LLPS into fibrillar center (FC), dense fibrillar center (DFC), and granular component (GC). Ribosomal DNA (rDNA) is transcribed into pre‐rRNA transcript by RNA polymerase I at the FC‐DFC boundary (see arrow A‐1). Key DFC‐GC proteins (FBL, NPM1) and ribosomal RNA (rRNA) are represented, with the big arrow showing the radial rRNA processing pathway followed by preliminary modification and cleavage in DFC. Fibrillarin (FBL), as part of the snoRNP complex, binds nascent pre‐rRNA transcript (A‐2) and catalyzes site‐specific 2′‐O‐methylation within the DFC, while additional snoRNPs mediate pseudouridylation. The modified transcript is subsequently cleaved and processed to the GC, where it assembles with ribosomal binding proteins (RBPs) into pre‐mature ribosomal subunits, with chaperone nucleophosmin (NPM1) facilitating their maturation (A‐3) prior to their export from the nucleolus (A‐4). These ribosomal subunits further mature within the nucleoplasm and then move out to the cytoplasm (A‐5) through the nuclear pore complex (NPC). (B) Nuclear speckles illustrating the interstasis mechanism where particular GA‐rich RNAs are sequestered by RBPs. The speckle is enriched in low‐complexity domain (LCD)‐containing SR proteins (shown as yellow circles), which concentrate via LLPS with GA‐rich mRNA (green) and provide a scaffold for RNA‐protein interactions. This enrichment of LCD‐containing proteins enhances binding specificity and promotes the dynamic organization of RNA within nuclear speckles. (C) SARS‐CoV‐2 viral ribonucleoprotein (vRNP) complexes, with a zoom showing ~600 nt long genomic RNA organized with approximately 12 nucleocapsid (N) protein molecules (~6 dimers). The multivalent interactions between N protein domains (NTD, IDR, CTD) and viral RNA drive the typical “beads‐on‐a‐string” morphology.

Interactions between RNAs and proteins promote the stabilization and regulation of biomolecular assemblies, such as viral complexes and biological condensates (Roden and Gladfelter 2021). Within the nucleolus, ribosomal (rRNA) and small nucleolar RNAs (snRNA) RNAs interact with intrinsically disordered proteins (IDPs), as well as multi‐domain proteins with both IDRs and stable structured domains, thereby forming the immiscible three phase membraneless FC‐DFC‐GC compartments (Feric et al. 2016; Mitrea et al. 2016). Furthermore, RNA‐protein interactions modulate the saturation concentration (*C*_sat_) and material properties (i.e., viscosity, surface tension, and selectivity) of the resulting condensates (Banerjee et al. 2017; Zhang et al. 2015). Importantly, this modulation is not monotonic in RNA content. Owing to their flexible, repetitive sequence motifs enriched in charged and aromatic residues, and their intrinsic propensity for LLPS, IDPs drive cooperative dynamic interactions that promote the assembly of biomolecular condensates (Martin et al. 2020; Wang et al. 2018). Additionally, sequence‐specific amino acid tracts, such as positively charged (arginine, lysine) or acidic disordered stretches, drive localization of biomolecular assembly and modulate biophysical properties of the resultant condensates and complexes (Nott et al. 2015; Pak et al. 2016). In particular, arginine‐rich low‐complexity domains (R‐LCDs) present in RBPs increase the formation of RNA‐protein derived nucleolar condensates (Chong et al. 2018). Similarly, the structural flexibility and sequence multiplicity of multivalent RNA molecules provide selective binding sites for proteins, altering the phase behavior of biomolecular condensates while contributing to their nucleation and stability (Maharana et al. 2018; Roden and Gladfelter 2021). Specific RNA motifs, for example, rRNA in the nucleolus and non‐coding RNA (NEAT1) in paraspeckles (Yamazaki et al. 2018), are the basic elements for RBP assembly, enabling specificity and dynamic regulation of meso‐scale condensate formation. The intrinsic sequence‐dependent conformational dynamics of RNA, as well as their ability to interact with partner proteins, determine their response to dynamic cellular signaling, thereby mediating crucial activities such as RNA processing and sequestration (Faraway et al. 2025; Van Treeck and Parker 2018). The effect of RNA depends on the RNA‐to‐protein stoichiometry, RNA length, and the balance between homotypic protein–protein, heterotypic protein‐RNA and homotypic RNA–RNA interactions, and it is characteristically nonmonotonic. Additionally, RNA length and stoichiometry set condensate surface tension and stability, and therefore their propensity to coalesce or persist as multiple droplets (Laghmach et al. 2022). Low‐to‐intermediate RNA levels can promote condensation, whereas excess RNA can suppress LLPS or generate alternative cluster‐like states (Alshareedah et al. 2019; Maharana et al. 2018). RNA is therefore best regarded as a tunable regulator of condensate formation and composition rather than solely as a promoter of phase separation.

To better understand these RNA‐protein interactions at the molecular level, mechanistic insight on their interplay is required in terms of sequence and structural determinants governing phase transitions as well as composition stability. Among the myriad cellular condensates, we will particularly focus this review on three paradigmatic RNA‐protein assemblies: nucleoli, nuclear speckles (NS), and viral ribonucleoprotein (vRNP) complexes. The rationale for this choice is that these three systems share a fundamental characteristic that distinguishes them from other membraneless organelles as they represent archetypal examples where RNA is not merely a passive client recruited to pre‐formed protein scaffolds, but rather serves as an active architectural element that directly nucleates, scaffolds, and modulates condensate formation and internal organization (Carlson et al. 2022; Faraway et al. 2025; Lafontaine et al. 2021). In the nucleolus, nascent rRNA recruits RBPs to establish the typical FC‐DFC‐GC organization (Lafontaine et al. 2021), while mRNA drives phase separation with RBPs in nuclear speckles (Faraway et al. 2025). vRNP complexes emerge as the most crucial in this regard, as the nucleocapsid protein must interact with genomic RNA to achieve both phase separation for transcription and subsequent condensation to pack the genome into new virus particles (Carlson et al. 2022). In this review, we will provide a critical discussion on RNA‐protein interactions and their role in the assembly and modulation of bio‐molecular condensates, their sequence‐dependent localization, the experimental and computational characterization, by focusing on (i) the nucleolus (Figure 1A), (ii) nuclear speckles (Figure 1B), and (iii) viral particles (Figure 1C).

## 2 Protein‐RNA Assemblies—Biophysical Principles

The interaction between RNA and protein regulates various cellular and patho‐physiological processes within diverse MLOs found in the human body (Banani et al. 2017; Lyon et al. 2021). We will elucidate how such interactions contribute to cellular processes including ribosomal processing and biogenesis within the nucleolus, sequestration of messenger RNA (mRNA) within nuclear speckles, as well as the role of RNA‐protein interactions in the viral assemblies (Figure 1).

### 2.1 The Nucleolus

The nucleolus, as a biomolecular condensate, serves as a fascinating model system for studying LLPS‐driven compartmentalization resulting from the interplay of RNA‐protein interactions (Boisvert et al. 2007; Lafontaine et al. 2021). Comprising of three functional subcompartments (FC‐DFC‐GC), it is responsible for the ribosomal biogenesis from rDNA to rRNA assembly and subsequent processing to the cytoplasm, where they translate into proteins (Hernandez‐Verdun et al. 2010; Pederson 2011). Within the nucleolus, each compartment has a distinct role, where the FC enables rDNA transcription by RNA polymerase I (Pol I) into rRNA, followed by early processing events of rRNA transcripts taking place on the FC‐DFC boundary (Yao et al. 2019). The rDNA transcription in the FC is regulated by a transcription factor, upstream binding factor (UBF), which binds rDNA repeats and facilitates the open chromatin state (Sanij et al. 2008). UBF works synergistically with multiple other nucleolar components including TATA‐binding proteins (TBP)‐containing selectivity factor 1 (SL1) that facilitate the transcription initiation by positioning Pol I at the rDNA promoter (Goodfellow and Zomerdijk 2012). Another IDR‐containing scaffold protein, TCOF1 (also known as treacle), promotes interactions between UBF and RNA Pol I to further support rDNA transcription (Calo et al. 2018). Resulting from the interplay of these nucleolar components, rDNA is transcribed into the 47S pre‐rRNA transcript at the FC‐DFC interface, with 5′ and 3′ external transcribed spacers (ETS) at both ends (Feric et al. 2016). The resulting transcript carries the sequences of the small‐subunit rRNA (18S), the large‐subunit rRNAs (5.8S and 28S) and the internal transcribed spacers (ITS), which are removed during rRNA processing to generate mature rRNA. The transition from FC to DFC is a critical event in the processing and modification of nascent rRNA, during which multiple small nucleolar ribonucleoproteins (snoRNPs) fold and catalyze site‐specific modifications, essential for proper ribosome function (Watkins and Bohnsack 2012). As the transcription proceeds, the nascent transcript, rich in negatively charged phosphate groups, immediately recruits positively charged arginine/lysine‐enriched IDR‐containing proteins, including fibrillarin (FBL) and nucleolar protein 58/56 (NOP56/58). The latter two proteins contain nucleolar localization signals (NoLS) motifs and form multivalent RNA‐protein interactions that drive condensation into the DFC (Yao et al. 2019). This interplay between the rRNA transcript and recruited IDPs forms an electrostatic and compositional gradient conceptually similar to the permeability barrier across the nuclear pore complex (NPC), where FG‐rich IDRs serve as selective filters for import and mediate regulated outflux (Frey and Görlich 2007; Schmidt and Görlich 2016). Both the compartments employ sequence‐driven, multivalent interactions to impose selectivity, create dynamic permeability barriers, and establish spatial compartmentalization for biochemical processing (Lafontaine et al. 2021; Riback et al. 2020). The resulting selective partitioning arises from active sorting processes where molecular crowding, macromolecular binding, and RNA‐protein interactions drive the radial outflux of rRNA from the nucleolus into the cytoplasm via the NPC (Quinodoz et al. 2025). The Box C/D and Box H/ACA are the main snoRNPs in the DFC responsible for 2′‐O‐methylation and pseudo‐uridylation, respectively (Watkins and Bohnsack 2012). Multiple nucleolar components in Box C/D, including FBL (Sun, Zhu, et al. 2024), NOP56, NOP58 (Dominique et al. 2024), small nucleolar ribosomal protein 13 (SNU13) (Dobbyn and O'keefe 2004) and, most importantly, U3 and U8 snoRNAs, are responsible for the 2′‐O‐methylation‐specific rRNA nucleotides (Quinodoz et al. 2025). U3 snoRNA promotes the 5′ ETS cleavage by base‐pairing with a specific region of the 5′ ETS and 18S rRNA that ultimately releases the 18S rRNA, forming the small subunit (SSU) (Phipps et al. 2011). Conversely, U8 snoRNA base pairs with the ribose group of the specific nucleotides in rRNA, and localizes them into the proximity of the key DFC protein, FBL, that modifies the relevant nucleotide and initializes large subunit (LSU) processing (Langhendries et al. 2016). On the other hand, Box H/ACA snoRNPs are essential for the pseudo‐uridylation of rRNA, stabilizing its structure (Ge and Yu 2013). Dyskerin (DKC1) is the key catalytic enzyme in Box H/ACA and is responsible for pseudo‐uridine synthesis (MacNeil et al. 2021). Likewise, other core proteins including glycine/arginine‐rich protein 1 (GAR1) (Félix et al. 2025), NHP2 and nucleolar protein 10 (NOP10) (Henras et al. 1998), are responsible for stabilizing the snoRNP complex and ensure correct folding of the corresponding RNA‐protein components. Pre‐ribosomal subunit assembly takes place in the GC subcompartment where further cleavage removes the ITS regions from the pre‐mRNA transcript, resulting in the mature 18S, 5.8S and 28S rRNAs (Henras et al. 2015). This is where proteins containing ribosomal binding domains (RBDs), including nucleophosmin (NPM1), bind to the processed rRNA and facilitates their assembly (Feric et al. 2016; Lafontaine et al. 2021). The PeBoW complex (composed of BOP1, PES1 and NPM3) and other core GC proteins, including SURF6 and RRP1, are responsible for the processing and maturation of ribosomal subunit assembly (Rohrmoser et al. 2007). The DEAD‐box helicase DDX18 also has a key role in RNA modeling during the transition of pre‐60S particles (Calo et al. 2015).

NPM1, in particular, emerges as a key protein in the GC that organizes and stabilizes other proteins and facilitates phase separation, critical for ribosome assembly (Feric et al. 2016; Mitrea et al. 2018). The multiphase nucleolar immiscibility has been reported to be driven by the interplay between NPM1 and FBL (also known as FIB1) (Feric et al. 2016). NPM1 (predominant in the GC phase) contains both structured oligomerization and ribosomal binding (RBD) domains and a central IDR enriched in charged residues (Mitrea et al. 2016, 2014). On the other hand, FBL, being abundant in the DFC phase, contains an extensive N‐terminal IDR and an RBD, necessary for the selective interactions with rRNAs (Yao et al. 2019). A study by Feric et al. reports that these two proteins, when interacting, have a tendency to replicate the typical DFC‐GC nucleolar architecture that is important for rRNA processing (Feric et al. 2016). High‐resolution imaging and biophysical characterization reveal that the NPM1 phase encapsulates the FBL phase (similar to the GC surrounding the DFC), generating sharp phase boundaries governed by the differential interaction affinities and interfacial tension. Mechanistically, this immiscibility results from the underlying inter‐ and intra‐molecular interactions between these two proteins, where the IDRs within both the proteins drive phase separation and the structured domains lead to the liquid–liquid immiscibility (Lafontaine et al. 2021). These inter‐domain interactions potentially are responsible to form thermodynamically‐stable compositionally discrete DFC‐GC phases, where the DFC phase is stabilized by FBL interactions and the GC phase by favorable NPM1 coacervation with rRNA and other proteins (Feric et al. 2016; Mitrea et al. 2016). The exact underlying molecular interactions and potential driving forces involved in nucleolar phase immiscibility, driven by FBL‐NPM1, are still poorly understood, which would be an interesting area to explore further.

Assembled in the GC phase, the pre‐40S and pre‐60S ribosomal subunits are then transported to the nucleoplasm for final maturation and quality control, prior to their export to the cytoplasm for protein synthesis (Nerurkar et al. 2015). The rRNA movement through the nucleolus is not merely by passive diffusion; it is an actively‐regulated highly ordered outflux pathway that is driven by the interplay of rRNA, processing factors and scaffolding proteins (Quinodoz et al. 2025). This mechanism acts as a radially controlled flux, from the inner FC to the DFC and then to the GC, and is tightly coupled to the rRNA maturation, compartmental phase properties, and the underlying molecular interactions. Importantly, existing studies have demonstrated that the rRNA processing throughout the nucleolus directly decides its progression outwards. For example, a recent study by Quinodoz et al. (2025) reports engineered nucleoli and perturbations of rRNA cleavage to show that unprocessed rRNAs accumulate at the DFC‐GC boundary without entering the GC phase (Quinodoz et al. 2025). This suggests that the rRNA processing significantly depends on biophysical changes, including altered molecular affinity and folding states, enabling its transition across phase boundaries. The cleavage of 5′ ETS sites (01/02) occurs at the FC‐DFC boundary within 0–15 min of 5‐ethynyl uridine (5eU) pulse labeling in a pulse‐chase experiment. Following this, ITS1 cleavage (1/2 sites) takes place within 30–60 min when rRNA moves from the DFC to the GC, and the late LSU cleavage (IT2) happens in the GC and then fluxes towards the nucleoplasm within 60–120 min (Quinodoz et al. 2025). This information suggests that the rRNA processing and its spatial progression opposite to the nucleolar periphery are correlated, with SSU maturation largely completing within the DFC, while LSU matures inside the GC. Further perturbation and knockdown of SSU processome components (U3 snoRNA or FBL) results in an “inside‐out” nucleolar morphology where FC‐DFC relocate to the periphery, trapping 18S precursors and early factors (ESF1, NAT10, NOP56, FBL) within the enlarged DFC, and ultimately reduces rRNA outflux (Quinodoz et al. 2025). On the other hand, LSU‐targeted perturbations (RPL5 or U8) slow the flux but the inside‐out inversion was not seen, suggesting a pathway‐specific coupling between rRNA maturation and phase organization. These results indicate that the RNA‐protein assemblies within the DFC form unprocessed intermediate rRNA that, upon irreversible changes, outfluxes to the GC and then to the nucleoplasm for final maturation. A study by Fine and Moses (2025) provides a physical framework of this behavior by concluding that the condensates maintain flux by cyclic condensation and their re‐mixing is promoted by the differences in free‐energy of mixing and additional interaction parameters, where electrostatic fields at condensate boundaries can localize charged polymers and bias their movement (Fine and Moses 2025). While RNA export to the cytoplasm is typically receptor‐mediated by nuclear transport receptors (NTRs), direct RNA‐protein interactions between rRNAs and nucleoporins, particularly FG‐repeat nucleoporins (FG‐Nups), have been proposed to facilitate selective passage through the nuclear pore complex (NPC) (Köhler and Hurt 2007; Li et al. 2023). Whether rRNAs are fully processed by NTRs or partially engage FG‐Nups via intrinsic interactions remains an active area of research.

### 2.2 Interstasis: How IDRs Sequester Their Own mRNA

In addition to rRNA processing, the concept of messenger RNA (mRNA) sequestration has recently emerged, followed by a comprehensive study by (Faraway et al. 2025). According to their findings, mRNAs are sequestered through the R‐MCD mediated accumulation of RBPs via a feedback mechanism termed interstasis, maintaining the overall intracellular homeostasis. This particular behavior results from the interplay between RBPs within the nuclear speckles, and purine‐rich (GA‐rich) multivalent mRNAs that encode condensation‐prone, arginine‐rich mixed‐charge domain (R‐MCD) proteins. This process is crucial for cellular equilibrium as it prevents the export of excess mRNA to the cytoplasm via the NPC, thereby controlling the synthesis of the corresponding proteins. The interstasis mechanism, in principle, is a hierarchical, concentration‐sensitive feedback loop. At its core, nuclear speckles serve as dynamic compartments where the balance between mRNA retention and export is actively‐regulated by the interplay between mRNA sequence composition, RBP concentration, and the accumulation of R‐MCD proteins (Faraway et al. 2025). Nuclear speckles are enriched in serine/arginine‐rich (SR) splicing factors, RNA helicases, and poly(A)‐binding proteins, and their material properties are modulated by the composition and concentration of their mRNA and protein constituents. Importantly, nuclear speckles exhibit liquid‐like behavior with rapid internal reorganization, as demonstrated by fluorescence recovery after photobleaching (FRAP) experiments (Kim et al. 2019), suggesting that sequestration and release of mRNAs are kinetically accessible processes rather than irreversible trapping events. Interestingly, codon‐biased multivalent mRNAs, with repetitive GA‐rich codons, promote interactions with nuclear speckle‐localized RBPs (such as the family of SR Serine/Arginine‐rich) proteins (Faraway et al. 2025; Shepard and Hertel 2009). It is important to note that although speckles are enriched in SR splicing factors, these are not individually required for speckle integrity. Systematic depletion experiments identified SON and SRRM2 as the organizing core, where loss of SON alone causes only partial disassembly, whereas co‐depletion of SON and SRRM2 results in near‐complete dissolution of speckles (Ilik et al. 2020). While these RBPs can independently trigger the selective capture of R‐MCD proteins encoded by GA‐multivalent mRNAs, their intermolecular interactions are further strengthened as the R‐MCD proteins accumulate, ultimately downregulating their translation process (Faraway et al. 2025). Furthermore, the RBPs (such as TRA2A, TRA2B) themselves, have the ability to interact and capture GA‐rich mRNAs within nuclear speckles, depending on their concentration. The purine‐rich codon bias is not incidental; GA‐rich sequences increase the density of potential RBP binding sites within mRNA, leading to the high multivalency. Faraway et al. reported that the higher dosage of RBPs alone can retain the mRNAs within the nuclear speckles, suggesting that the R‐MCDs are crucial when the RBPs are present in a moderate amount (Faraway et al. 2025). Likewise, this dosage sensitivity is applicable to R‐MCD proteins, meaning that their moderate concentrations have a low tendency to promote the cohesion of nuclear speckles. In principle, a bistable‐like regime is present within the nuclear speckles, where the concentration below a certain threshold leads mRNA to be efficiently exported to cytoplasm, and above threshold concentration of both effectors (RBPs) and mediators (R‐MCDs) drive mRNA sequestration. This switch‐like behavior is consistent with the thermodynamic principles of LLPS, where small changes near the *C*_sat_ induce large effects on condensate stability (Li et al. 2026). This overall selectivity is sequence‐encoded, with purine rich mRNAs more likely to be sequestered, compared to the pyrimidine‐rich mRNAs. This might be due to their intrinsic properties including increased base stacking, higher charge density, and their structured potential for multivalent cation‐π and electrostatic interactions (Vernon et al. 2018; Wang et al. 2018). Another recent study by Xu et al. reported that the increased glycine concentration (0.3–0.9 mM) supresses NPM1‐rRNA phase separation within the nucleolus, affecting their condensation dynamics (Xu et al. 2025). This is in accordance with Faraway et al. that show that the tendency of droplet formation via LLPS is concentration‐dependent. Notably, the glutamate inclusion to the NPM1‐rRNA condensate were found to promote their phase separation, which aligns with the previous studies that the specific sequence composition can modulate the phase behavior, either by suppressing or by promoting their underlying intermolecular interactions (Kaur et al. 2021; Xu et al. 2025).

### 2.3 RNA‐Protein Interactions in Viral Assembly

In addition to biomolecular condensates, viral assemblies have been reported to be influenced by the interplay between RNA and proteins (Iserman et al. 2020; Savastano et al. 2020). SARS‐CoV‐2, a paradigmatic single‐stranded positive‐sense RNA virus, is a fundamental example of this phenomenon (V'kovski et al. 2021). With over 30 kb genome size, it is packaged within compact, pleomorphic ribonucleoprotein (vRNP) complexes, where 30–40 vRNPs per virion form discrete particles rather than an extended continuous shell (Klein, Cortese, et al. 2020; Yao et al. 2020). This is consistent with the LLPS phenomena, where membraneless liquid‐like droplets are formed within certain organelles (including the nucleolus) (Cubuk et al. 2021). More specifically, LLPS of N protein with RNA may contribute to transcriptional or assembly intermediates, whereas mature virions contain discrete, structurally organized vRNP complexes (Carlson et al. 2022; Klein, Cortese, et al. 2020). A study by Carlson et al. reports that each vRNP is arranged via the interplay between 12 nucleocapsid N proteins (6 dimers) and 600‐nucleotide‐long viral RNA (Carlson et al. 2022), see Figure 1C. This RNA has been reported to be potentially the part of multiple genomic regions, which indicate that there is no specified sequence of RNA that is supposed to bind with the N protein dimers (Carlson et al. 2022; Iserman et al. 2020). Furthermore, even small fragments of viral RNA have the ability to trigger vRNP formation, suggesting that 600‐nt long RNA is not always required to form ribonucloesomes (Carlson et al. 2022). This sequence tolerance suggests that the driving force for vRNP assembly is not sequence‐specific recognition, but rather a combination of electrostatic complementarity between the negatively charged RNA phosphate backbone and the positively charged RNA‐binding domain of N protein. Apart from two short N‐ and C‐terminal disordered patches, the SARS‐CoV2 N protein consists of three primary domains including an N‐terminal RNA‐binding domain (NTD), a dimerizing C‐terminal domain (CTD) and a central extensive IDR that connects both the NTD‐CTD domains, and that is potentially critical in phase separation (Cubuk et al. 2021; Jack et al. 2022) (Figure 1C). Importantly, SR‐rich central IDR undergoes phosphorylation‐dependent conformational changes that shape the transition between LLPS‐competent and genome‐packaging state (Carlson et al. 2022). Specifically, hyperphosphorylation of the IDR by host kinases SR protein kinase 1 (SRPK1) and casein kinase II (CK2) reduces its interaction with RNA and promotes the liquid‐like, transcription‐competent state (Bouhaddou et al. 2020; Stuwe et al. 2024), while hypophosphorylated N protein preferentially forms the compact ribonucleosomal structures associated with genome packaging (Carlson et al. 2022). This phosphorylation switch is a key regulatory mechanism by which virus can transit between transcription and packaging modes within the same host cell. When mixed with viral RNA, the N proteins form higher‐order viral assemblies in vitro via multivalent protein‐RNA interactions (Perdikari et al. 2020; Savastano et al. 2020). Interestingly, leucine‐rich helix (LH) regions within the central IDRs of N proteins are crucial for self‐association, which is in correspondence with the role of arginine/lysine rich DFC‐GC partitioning in the nucleolus (Adly et al. 2023; Carlson et al. 2022). Mechanistically, the RNA secondary structure has a dominant control over the resultant biomolecular assemblies, with short stem‐loop (SL) RNAs from 5′ genomic regions promoting the compaction of the flexible N dimers to the ordered units (Iserman et al. 2020). On the other hand, IDRs facilitate the nucleation and ordering of the corresponding assemblies, reflecting the overall sequence‐specific partitioning of nuclear condensates (Cubuk et al. 2021). It has also been reported that the specific RNA motifs serve as selective anchors that actively recruit and cluster the N molecules (Iserman et al. 2020). These motifs are crucial in encoding multivalency and localization, necessary for viral assembly. This is similar to the role of rRNA, snoRNA, or long noncoding RNA (lncRNA) in the nucleation and phase separation of nucleolar and nuclear speckle condensates (Roden and Gladfelter 2021; Van Treeck and Parker 2018). Overall, by packaging the whole viral genome into the constrained spaces and encoding biomolecular assemblies, vRNPs mimic the principles of phase separation and selective partitioning.

## 3 Experimental and Computational Characterization

### 3.1 Biophysical and Imaging Techniques

A combination of emerging biophysical techniques and high‐resolution imaging have advanced the experimental and computational characterization of RNA‐protein assemblies in recent years (Alberti et al. 2025; Mittag and Pappu 2022) (Figure 2). Because RNA‐protein assemblies are architecturally hybrid (i.e., combining folded domains, extended IDRs, and structurally polymorphic RNA within a single particle), no single technique reports on all of their structural features, and the available methods differ systematically. We therefore group them into (i) live‐cell imaging and rheological approaches that report on condensate‐scale dynamics and material properties, (ii) solution‐scattering and spectroscopic approaches that are related to ensemble‐averaged, low‐resolution conformational information and constitute the primary source of experimental restraints for disordered regions, and (iii) high‐resolution imaging approaches that resolve folded domains and ordered assemblies at near‐atomic detail but are largely blind to the disordered segments that frequently drive phase separation.

**FIGURE 2:**
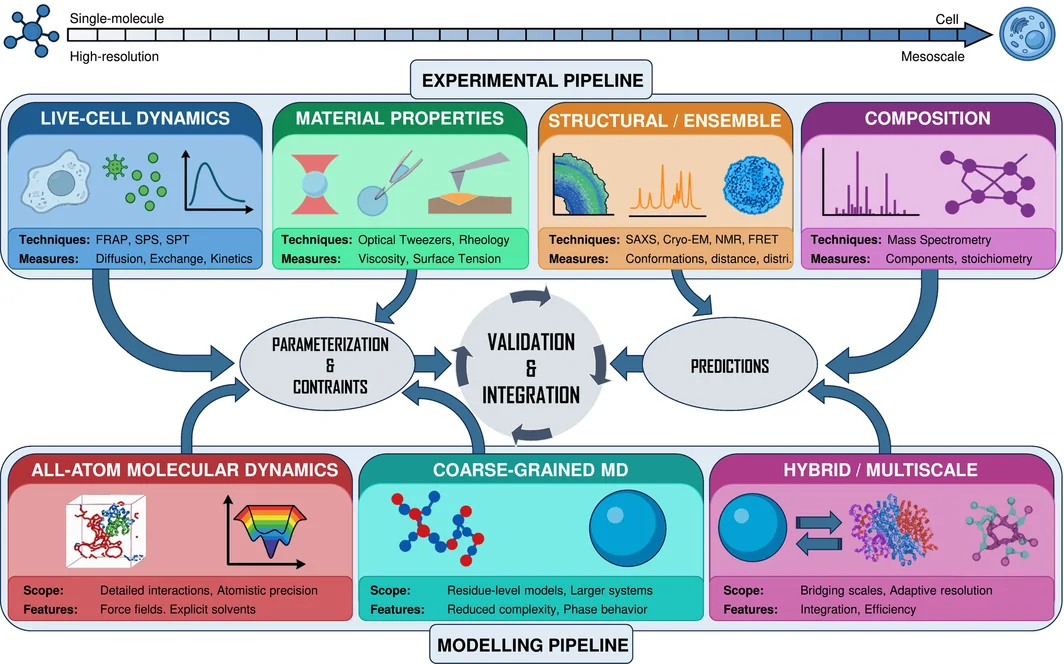
An integrated experimental and computational pipeline for multi‐scale condensate characterization. The figure illustrates the integration between multi‐scale experimental observations (top) and physics‐based modeling (bottom). (Top) Experimental characterization via various techniques available: Live‐cell dynamics (e.g., fluorescence recovery after photobleaching (FRAP), single‐particle spectroscopy (SPS), single‐particle tracking (SPT)) quantify diffusion, molecular exchange, and kinetic heterogeneity within condensates. Material properties are probed via optical tweezers and microrheology to measure viscoelasticity, surface tension, and fusion behavior. Structural/ensemble techniques, including SAXS (small‐angle X‐ray scattering), SANS (small‐angle neutron scattering), NMR (nuclear magnetic resonance), Cryo‐EM (cryo‐electron microscopy), and FRET (Förster resonance energy transfer), explore conformational states, molecular organization, underlying interactions, and distance distributions. Composition is assessed via mass spectrometry to determine molecular components. (Center) Experimental data forms a foundation for computational parameterization and modeling, creating a validation loop where computational predictions are benchmarked against experimental observables. (Bottom) The modeling strategies are applicable at different levels of resolution. Complex all‐atom molecular dynamics can capture explicit solvent and atomic‐level interaction patterns, whereas residue‐level coarse‐grained models are used to uncover mesoscale phase behavior. Hybrid approaches couple the all‐atom and coarse‐grained modeling approaches for efficient simulations while preserving atomic‐level accuracy.

#### 3.1.1 Live‐Cell Imaging and Material Properties

In general, the high‐resolution time‐lapse imaging techniques have been most instrumental for direct observation of condensate forms, fusions and dissolution under physiological and stress conditions (Taylor et al. 2019). The correlation between the phase behavior and functional characterization can be observed and quantified using the different strategies lying within the high‐resolution time‐lapse imaging. A recent study by Wu, King, et al. (2025); Wu, Wang, et al. (2025) explored the submicrometric arrangement of RNA‐protein condensates via super‐resolution imaging alongside environmentally sensitive fluorogens, visualizing the condensed states on a 1000–100,000 times scale smaller than that of the width of a human hair (Wu, King, et al. 2025). Their findings revealed that the condensates are not simple and uniform droplets, but possess shifting, network‐like molecular organizations. Likewise, confocal fluorescence microscopy has been widely utilized to probe the spatial organization and dynamic behavior of the RNA‐protein assemblies in nucleolar and viral cells (McClelland et al. 2021; Richter et al. 2025). A study by Acharya et al. (2025) employed label‐free fluorescence microscopy to uncover a distinct property of biomolecular condensates (Acharya et al. 2025). Without using any external fluorescent label, they directly visualized condensate formation, its core‐shell architecture, and dynamic molecular exchange, demonstrating that many biomolecular condensates exhibit intrinsic fluorescence (Acharya et al. 2025). Another study combined single‐photon detection with time‐lapse super‐resolved imaging to track the molecular mobility, intermolecular interactions, and nano‐environmental properties within nuclear speckles (Perego et al. 2023). The integration of fluorescence microscopy with the FRAP enable the quantitative measurement of underlying material properties, including viscosity and internal mobility, of biological assemblies (Ng et al. 2025; Santamaria et al. 2024). Recently, the distinct mechanical properties of biomolecular condensates, such as surface tension and viscosity, have been efficiently measured by combining fluorescence microscopy with atomic force microscopy (AFM) in a single experiment, which was termed “fluorescence recovery after probe‐induced dewetting” (FRAP‐ID) (Santamaria et al. 2024). Crucially, the physical manipulation of individual droplets at the single‐molecular assembly level can be enabled by the use of optical tweezers, the instruments using highly focused laser beams to control the movement of biological components (Bustamante et al. 2021). Yuzu et al. (2024) induced and even spatiotemporally controlled the formation of biological condensates at an air/solution interface via optical tweezers, revealing new details about material heterogeneity and molecular organization (Yuzu et al. 2024). Moreover, the applications of dual‐trap optical tweezer allow a quantitative examination of the fusion properties and rheology of ternary RNA‐protein condensates, demonstrating the tunable material properties of these biomolecular assemblies which are dependent on their molecular composition (Mahendran et al. 2025).

#### 3.1.2 Ensemble Methods for Disordered Regions

*Ensemble‐averaged* structural properties (such as overall shape, size, and conformational flexibility) of biomolecular assemblies are widely probed using solution‐scattering techniques such as Small‐Angle X‐ray Scattering (SAXS) and Small‐Angle Neutron Scattering (SANS) (Borthakur et al. 2025; Da Vela and Svergun 2020). These are low‐resolution methods as the measured intensity is ensemble averaged, which makes them well suited for flexible and intrinsically disordered systems (Cordeiro et al. 2017; Kikhney and Svergun 2015). SAXS/SANS therefore report on the distribution of conformations sampled by IDRs and disordered RNA‐protein complexes, typically through radius of gyration (*R*_*g*_), maximum dimension *D*_max_, and the pair‐distance distribution p(r) (Cordeiro et al. 2017; Kikhney and Svergun 2015). SAXS and SANS are among the most dominant benchmarks for testing and validating computational results in recapitulating experimentally observed structural distributions. As SAXS is highly sensitive to the changes in crowding, it can reveal both the solid‐like and liquid‐like structural organization without requiring crystallization or labeling protocols (Mohammed et al. 2024). Recently, research by Wang, Brasnett, et al. (2025); Wang, Hu, et al. (2025) utilized contrast‐variation SAXS (CV‐SAXS) to capture the molecular grammar, including base stacking and backbone extension, within the conformations of poly‐U, poly‐C, and poly‐A RNA condensates, assembled with pathologically relevant poly‐(proline‐arginine) (PR) peptides (Wang, Hu, et al. 2025). Furthermore, the development of time‐resolved SAXS (TR‐SAXS) and size‐exclusion chromatography coupled SAXS (SEC‐SAXS) techniques have improved the analysis of transient or heterogeneous assemblies by analyzing how they form, grow and rearrange in real‐time at the nanometer scale (Brosey and Tainer 2019). Unlike SAXS, SANS allows the contrast‐matching of biological components, such as deuterated (D‐labeled) RNA and proteins, enabling the direct interrogation of the individual molecular species within complex biomolecular assemblies (Krueger 2022). An integrated SANS‐FRAP strategy was recently utilized to characterize nucleolar condensates, mimicking a GC morphology. Through this experiment, it was found that these condensates form “network fluids” with pronounced spatial inhomogeneities, rather than simple homogeneous liquids (Dar et al. 2024). The trade‐off of this ensemble sensitivity is that scattering data alone cannot resolve residue‐level contacts or the internal architecture of folded domains: the information content of a one‐dimensional scattering curve is insufficient to determine a unique three‐dimensional structure, so SAXS/SANS are most powerful when interpreted through ensemble‐modeling approaches or combined with high‐resolution restraints (Cordeiro et al. 2017; Da Vela and Svergun 2020). Similarly, nuclear magnetic resonance (NMR) spectroscopy is arguably the most informative technique for disordered regions, since it reports at residue resolution on secondary‐structure propensity, backbone dynamics and transient contacts that are averaged out in scattering data. As chemical shifts and paramagnetic relaxation enhancements (PREs) are among the experimental restraints that are widely used to construct and validate IDR ensembles, high viscosity of the dense phase makes such measurements demanding. Therefore, the direct NMR inside condensates is now well established. For instance, solution NMR of phase‐separated FUS‐LCD showed that it remains disordered within the liquid state and engages only transient intermolecular contacts (Burke et al. 2015), and equivalent measurements on Ddx4 droplets recovered sharp resonances and quantified the reduced but persistent backbone mobility of the condensed phase (Brady et al. 2017). Similarly, a recent work has systematically compared IDP dynamics between the dilute and condensed states, showing that phase separation slows internal motions in a residue‐dependent manner (Guseva et al. 2023). Within the paradigmatic systems reviewed here, NMR has resolved the multi‐modal interactions through which NPM1 engages R‐rich linear motifs and rRNA in the nucleolar GC (Mitrea et al. 2016), and has shown that phosphorylation of the SR‐rich region of the SARS‐CoV‐2 N protein regulates phase separation by inhibiting self‐association of a distant helix (Stuwe et al. 2024). On the other hand, Förster resonance energy transfer (FRET) reports on structural properties, providing intramolecular distances and their distributions for disordered chains. FRET combined with SAXS established that the central IDR of the SARS‐CoV‐2 N protein remains expanded and dynamic even in the RNA‐bound state (Cubuk et al. 2021). Importantly, this approach is now applicable in situ, where Yu et al. (2023) measured distance distributions for 19 labeled segments of the FG domain of NUP98 with functional NPCs using site‐specific non‐canonical amino acid labeling together with fluorescence‐lifetime imaging FRET. Their results showed that the FG domain experiences good‐solvent conditions and adopts far more extended conformations inside the assembled NPC than isolated chains in solution (Yu et al. 2023). Since FG‐Nups are among the best‐characterized disordered assemblies, such in‐cell measurements provide a direct benchmark for CG models of IDR assemblies.

#### 3.1.3 High‐Resolution Methods for Ordered Assemblies

Complementary to these ensemble methods, and with an essentially opposite scope, cryogenic electron microscopy (Cryo‐EM) resolves the folded and ordered components of RNA‐protein assemblies at near‐atomic resolution. Because cryo‐EM maps are reconstructed by averaging many particle images, rigid and conformationally homogeneous regions are resolved most clearly, whereas flexible segments are often blurred or absent from the final density (Nwanochie and Uversky 2019). Cryo‐EM is therefore well suited to structured domains, while flexible IDRs often remain poorly resolved. Combined with cryo‐electron tomography (cryo‐ET) (Klein, Cortese, et al. 2020), cryo‐EM provides structural insights into SARS‐CoV‐2 virions and identifies the vRNPs organization within viral assemblies. The cryo‐EM's ability to capture these organizations at high‐resolution opens the opportunity to explore how N protein forms distinct RNA‐binding complexes with the viral genomes, which is crucial for genome compaction and assembly into viral particles. Another study (Carlson et al. 2022) reconstituted vRNP complexes in vitro, and used cryo‐EM to resolve their structural organization, providing mechanistic insights into viral genome packaging into the nucleocapsid. The SARS‐CoV‐2 vRNP illustrates this complementarity particularly well. Cryo‐ET of intact virions resolves the compact, cylindrical ~14–15 nm vRNP densities and their spatial arrangement within the nucleocapsid (Klein, Cortese, et al. 2020), and single‐particle reconstructions resolve the globular NTD and CTD of the N protein. However, neither approach resolves the central SR‐rich IDR whose phosphorylation state governs the switch between the LLPS‐competent and the genome‐packaging state. Conformational information on the disordered region has instead come from ensemble and single‐molecule measurements (Cubuk et al. 2021). Therefore, cryo‐EM and cryo‐ET are strictly complementary, and it is their combination that provides the restraints required to parameterize and validate models of the complete assembly.

Nevertheless, these experimental strategies have multiple distinctive shortcomings that limit their utilization. Fluorescence microscopy, for example, has certain difficulties to resolve the molecular‐scale heterogeneities inside condensates due to their fragile readouts (Alberti et al. 2025), and photobleaching (Lorenzetti et al. 2025). A recent study by Mohammed et al. (2024) demonstrates that the SAXS measurements are particularly sensitive to sample polydispersity, indicating the limited resolution and interpretability of the scattered data (Mohammed et al. 2024). Another study by Straub et al. (2025) explained how laser flash melting and conventional revitrification of cryo‐EM samples can trap the particles in a non‐equilibrium angular distribution, thereby reducing the preferred orientation that further limits the experimental reliability (Straub et al. 2025).

### 3.2 Computational Modeling

To overcome these limitations, physics‐based computational methods—particularly molecular dynamics (MD)—have emerged as important alternatives due to their efficiency and reduced computational costs (Saar et al. 2023; Schäfer and Stelzl 2025). Atomistic simulations can explore chemical driving forces including solvation thermodynamics, ion and small‐molecule partitioning, interfacial water structure, residual secondary structure, hydrogen bonding, and (most importantly) specific RNA‐protein contacts that are otherwise inaccessible to imaging or ensemble scattering (Best 2017; Robustelli et al. 2018). Direct atomistic simulations of condensed phases are also feasible at more limited system sizes and timescales. For example, microsecond explicit‐solvent simulations of FUS‐LC and LAF‐1 RGG condensates resolved residue‐level intermolecular contacts, chain conformations, ion and water partitioning, and condensed‐phase molecular dynamics (Zheng et al. 2020). Such simulations provide atomic‐scale information within an established dense phase, although direct equilibration of mesoscale phase coexistence remains computationally demanding. One of the major downsides of all‐atom MD simulations is the high computational cost, especially when dealing with larger biological systems, similar to the biomolecular assemblies (Kmiecik et al. 2016). Many conventional atomistic forcefields are optimized for folded proteins, and can therefore inadequately describe the flexibility of the disordered RNA/protein molecules and their interaction with the solvent (Zerze et al. 2019). ABSINTH, an atomistic forcefield, employs an implicit solvent strategy while efficiently capturing the conformational dynamics of IDRs and nucleic acids (Vitalis and Pappu 2009). Although this model indeed reduces the computational cost and increases efficiency by treating solvent effects via a combined mean‐field electrostatic potential and solvation free energies, the incorporation of all atoms remains expensive when modeling complex biological events, such as nucleation and phase separation. Despite being reliable for single‐molecule or small‐chain calibration, all‐atom MD simulations are yet unsuitable for mesoscale condensate equilibria, and thus alternative computational approaches are needed to bridge these barriers (Dignon et al. 2018).

Coarse‐grained (CG) simulations have recently transformed biomolecular condensate research due to their computational efficiency and efficacy to model phase separation phenomena (Dignon et al. 2018; Joseph et al. 2021; Rizuan et al. 2026) (Figure 3 and Table 1). CG modeling manages to minimize computational cost by simplifying molecular representations and clustering groups of atoms into larger representative “pseudo‐atoms” or “beads”. Such adaptations facilitate the modeling of complex biological systems, such as biomolecular assemblies, for longer timescales with reduced computational expense, by smoothing out atomistic details at one end while preserving crucial structural information at the other (Dignon et al. 2018; Joseph et al. 2021; Kmiecik et al. 2016). Biological systems, such as protein‐RNA condensates, can be modeled by two CG approaches: bottom‐up and top‐down approaches. Bottom‐up analyses derive CG coordinates directly from atomistic data, reducing the degrees of freedom in such a way that the resultant representations are ensured to reproduce the structural and thermodynamic characteristics of the corresponding all‐atom system (Jin et al. 2022). Top‐down approaches, on the other hand, consider experimental data as a benchmark dataset to derive a compatible CG forcefield that captures a representative quantity, such as radius of gyration (*R*_*g*_), hydrodynamic radius (*R*_*h*_), or phase boundaries (Latham and Zhang 2019; Tesei et al. 2021). Interestingly, the detailed accuracy of bottom‐up derivations and the transferability of top‐down optimization led to numerous hybrid CG forcefields, improving applicability across various biomolecular systems (Liebl and Voth 2025; Shireen et al. 2022). Through iterative refinements, integration of diverse datasets, and their validation against both experimental as well as atomistic data, existing CG models have been optimized to capture the relation between sequence, structure, and environment of RNA‐protein assembly formation and dynamics (Cao et al. 2024; Joseph et al. 2021).

**FIGURE 3:**
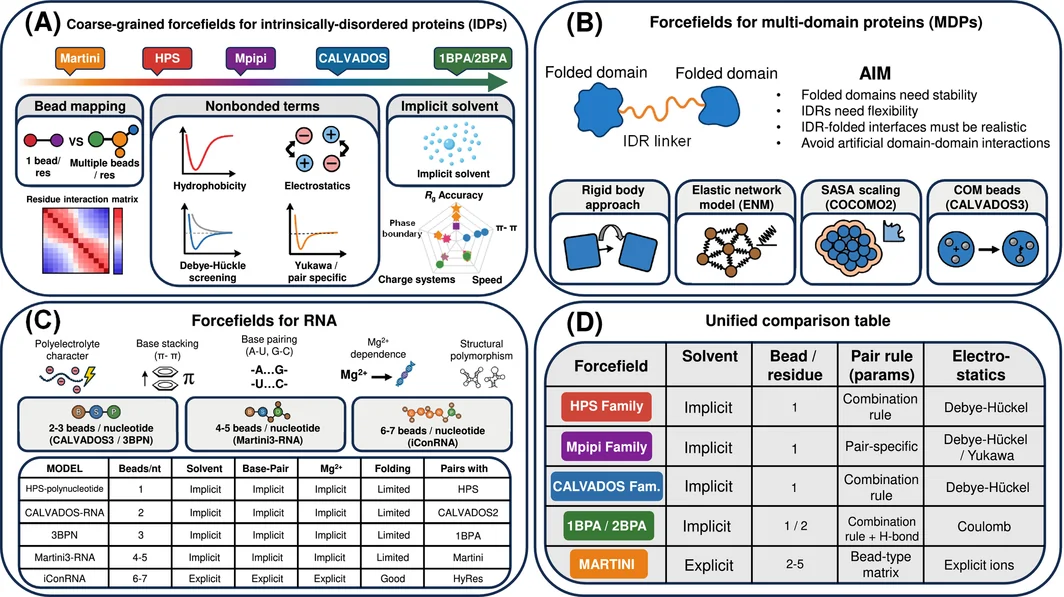
Coarse‐grained modeling frameworks for RNA‐protein complexes. (A) Panel A shows a variety of existing CGIDP forcefields, based on their bead‐mapping (ranging from one‐bead to multi‐bead), and non‐bonded interactions. HPS and CALVADOS utilize hydrophobicity scales with Debye‐Hückel electrostatics, while Mpipi prioritizes π‐π and cation‐π interactions via pair‐specific potentials. (B) Multiple strategies to accurately handle MDPs are shown. Among them, rigid‐body and elastic network model (ENM) to preserve folded regions, surface area scaling (COCOMO2) to modulate burial, and center‐of‐mass (COM) representation (CALVADOS3) to prevent over‐compaction. (C) Representation of various RNA‐specific forcefields that model RNA based on varied bead‐mapping (from 2–3 beads/nt to 6–7 beads/nt). (D) A comparative summary of key forcefield families, discussing their technical‐framework map.

**TABLE 1:** Summary of representative residue‐level CG forcefields to model RNA‐protein interactions in biomolecular assemblies. Herein, models are grouped by solvent treatment and bead mapping, then by the form of the short‐range non‐bonded term and its pair rule, since these choices determine the capabilities and limitations of a model independently of the datasets used for parameterization.

Forcefield	Mapping and solvent	Short‐range interaction framework	Electrostatics	Folded domains	Current scope
HPS (Dignon et al. 2018)/HPS‐Urry (Regy et al. 2021)	1 bead/residue, implicit solvent	AH; *λᵢj* = (*λᵢ* + *λj*)/2	DH	Rigid body	Highly efficient; a mixing rule cannot encode pair‐specific aromatic chemistry
MOFF (Latham and Zhang 2019)	1 bead/residue, implicit solvent	Pair‐specific *ε*_*ij*_ matrix, maximum‐entropy optimized	DH, distance‐dependent dielectric	Folds natively (energy‐landscapes)	Both folds globular proteins and reproduces IDP *R*_*g*_; single‐chain trained
MPIPI (Joseph et al. 2021)/MPIPI‐recharged (Andrés et al. 2025)	1 bead/residue, implicit solvent	Pair‐specific WF potentials informed by atomistic PMFs	DH/pair‐specific non‐symmetric Yukawa	Restraints	Correct ranking of π‐π and cation‐π; larger parameter space to transfer
CALVADOS v1 (Tesei et al. 2021)/v2 (Tesei and Lindorff‐Larsen 2023)	1 bead/residue, implicit solvent	AH; arithmetic‐mean λ, Bayesian‐optimized	DH, T‐dependent dielectric	ENM	Broad transferability across sequence and salt; similar mixing‐rule limitation as HPS
CALVADOS 3 (Cao et al. 2024)	1 bead/residue, implicit; COM + ENM	AH; arithmetic‐mean λ	DH	ENM + COM mapping	COM mapping removes the *C*_α_ side‐chain‐exposure artifact for MDPs
Martini3‐IDP (Wang, Brasnett, et al. 2025)	x, explicit solvent + ENM	LJ over a chemical bead‐type matrix	Explicit ions, Coulomb	ENM/Gō	Gives solvent‐mediated interactions and dynamics directly; high sampling cost, sequence specificity averaged into bead types
1BPA (Dekker et al. 2023; Driver and Onck 2025; Ghavami et al. 2014)	1 bead/residue, implicit solvent	LJ‐type; residue‐specific hydrophobicity	Coulomb, screened	ENM	Backbone statistics are residue‐resolved; no pair‐specific chemistry
2BPA (Adupa et al. 2026; Dekker et al. 2026; Dekker, van der Klok, et al. 2025)	2 beads/residue, implicit solvent	LJ‐type; residue‐specific hydrophobicity	Coulomb, screened	Emergent alpha and β‐sheet order	Extra beads enable β‐sheet and order‐to‐disorder transitions
CALVADOS‐RNA (Yasuda et al. 2025)	2 beads/nt, implicit solvent	AH; hydropathy‐based	DH (monovalent)	Pairs with CALVADOS	Disordered RNA and non‐specific sequence‐independent protein‐RNA LLPS
3BPN (Driver et al. 2024; Holmstrom et al. 2019)	3 beads/nt, implicit solvent	KR‐scale optimized	DH (monovalent)	Pairs with 1BPA	Coarse RNA folding details
HPS‐polynucleotide (Regy et al. 2020)	1 bead/nt, implicit solvent	AH; nucleotide hydropathy parameters with arithmetic mixing	DH (monovalent)	Pairs with HPS	Protein‐polynucleotide LLPS; no stacking or base‐pairing terms
COCOMO2 (Jussupow et al. 2025)	1 bead/residue, implicit + ENM + SASA	LJ‐type with SASA‐scaled *ε*	DH	ENM + SASA scaling	One scheme for folded + disordered + RNA; scaling is geometric, not chemical

Abbreviations: AH, Ashbaugh‐Hatch; COM, center‐of‐mass; DH, Debye‐Hückel; ENM, elastic network model; IDP, intrinsically disordered protein; KR, Kapcha‐Rossky; LJ, Lennard‐Jones; LLPS, liquid–liquid phase separatio; nt, nucleotide; SASA, solvent‐accessible surface area; WF, Wang‐Frenkel.

Despite their reduced computational cost, CG models can still face substantial sampling limitations when simulating phase separation. Slow exchange between dense and dilute phases, finite‐size effects, and delayed relaxation within concentrated phases can hinder convergence, particularly when estimating low saturation concentrations (Feito et al. 2025). Direct‐coexistence slab simulations partly alleviate these limitations by sampling the dense and dilute phases simultaneously and reducing finite‐size artifacts. Alternative Monte Carlo and lattice‐based enhanced‐sampling approaches can further improve exploration of configuration space, while GPU‐accelerated simulations extend the accessible time and length scales (Alberti et al. 2025). Nevertheless, careful convergence tests remain essential for reliable phase‐diagram and condensate‐property predictions.

Among the discussed approaches, only the residue‐level CG models currently reach the length and time scales of condensates‐level RNA‐protein assemblies while retaining sequence specificity. The following section therefore examines recent developments in CG forcefields for IDRs, MDPs, and RNA, with clear emphasis on their underlying representations, parameterisation strategies, capabilities and limitations.

## 4 Advances in Coarse Grained Forcefields

### 4.1 Modeling Intrinsically Disordered Proteins (IDPs)

The low computational cost and high efficiency of CG simulations have led to the development of multiple forcefields that can accurately account for hundreds of IDRs replicating phase separation phenomena of protein and RNA‐protein assemblies (Dignon et al. 2018; Ruff et al. 2019). These models typically use residue‐level representations with an implicit‐solvent strategy, so that explicit water molecules are excluded but their average thermodynamic effects, including ionic and dielectric screening, hydrophobic interactions, and solvation forces remain included via effective mean‐field potentials (Dignon et al. 2020). These models are best distinguished by the underlying technical choices, including the bead mapping, solvent representation, short‐range interaction potentials, and electrostatic treatment. For instance, explicit‐solvent and multi‐bead models provide greater structural and solvent‐mediated detail but at higher computational cost, whereas one‐bead‐per‐residue models offer greater sampling efficiency. Another important distinction is whether pair interactions are generated by residue‐specific parameters via combining rules, or by pair‐specific interaction matrices. Table 1 and Figure 3 are organized according to these framework‐level differences.

#### 4.1.1 Martini

The Martini forcefield, one of the initial top‐down CG models, was developed from seven bead classes including P (polar), N (intermediate/non‐polar), C (apolar), X (halo‐compounds), Q (monovalent ions), D (divalent ions), and W (water), wherein each class splits into multiple subtypes and each subtype is further categorized into regular (R), small (S), and tiny (T) sizes to modulate packing and interaction strengths (Marrink et al. 2007; Marrink and Tieleman 2013). Martini‐3, an advanced version of Martini, has formalized a combined parameterization strategy, with non‐bonded interactions being modeled via a top‐down approach and bonded potentials through a bottom‐up formulation (Souza et al. 2021). Given the emergence of phase separation phenomena, a Martini‐IDP variant has been recently introduced to model the interactions between multidomain proteins, including both the structured and unstructured regions (Wang, Brasnett, et al. 2025). This involves the extension of the original Maritni‐3 model by keeping the same non‐bonded interaction matrix but retuning the bonded parameters to match the IDP conformational ensemble with the available atomistic and experimental data. While Martini variants provide a chemically intuitive CG description, their complexity and bead hierarchy make them relatively heavy and less directly tunable for sequence‐driven phase behavior.

#### 4.1.2 HPS

In this regard, one‐bead‐per‐residue models (e.g., the hydropathy scale (HPS) family) can potentially be more efficient, with each residue being represented by only one bead, significantly reducing the overall computational cost (Dignon et al. 2018; Ghavami et al. 2014). HPS (hydropathy scale) models are best described as empirical, knowledge‐based residue‐level models rather than conventional bottom‐up models. Their residue‐residue interactions are not systematically derived from atomistic reference data, but are assigned from tabulated hydropathy scales and combined with polymer‐physics functional forms, so that the underlying chemistry enters only through the choice of scale (Dignon et al. 2018). Generally, each residue in HPS is modeled via the corresponding backbone *C*_α_ bead, with the short‐range non‐bonded interactions being defined by the hydropathy‐dependent modified Ashbaugh‐Hatch potential (Ashbaugh and Hatch 2008), and the electrostatics are set via a Debye‐Hückel term (Ullner et al. 1996) with implicit solvent and salt. Although the initial HPS Kapcha‐Rossky (HPS‐KR) model was substantially improved over atomistic hydropathy scales, it is often limited in reproducing quantitative experimental trends, such as phase separation upon mutations, which led to the development of HPS‐Urry (Regy et al. 2021; Urry et al. 1992). The updated HPS‐Urry model, derived from elastin‐like peptides, is considered to be closer to the experimental observations of protein assemblies both quantitatively and qualitatively because the Urry scale supplies a better‐calibrated set of *λ* values together with two adjustable parameters that rescale and shift it. HPS‐Urry does not introduce a separately parameterized cation‐π term, and such effects are represented only implicitly through the effective residue‐residue interactions (Regy et al. 2021). Likewise, HPS‐secondary structure (HPS‐SS) model further introduced the sequence‐specific bonded and dihedral potentials that reproduce transient secondary structure (especially helices) seen in atomistic simulations and experiments, while keeping the same hydropathy‐based non‐bonded interactions (Rizuan et al. 2022). Overall, the HPS models are computationally efficient, conceptually simple, and qualitatively accurate but they often mis‐rank the relative importance of π‐π interactions and potentially miss quantitative phase boundaries, especially for highly charged domains (Das et al. 2020; Regy et al. 2021).

#### 4.1.3 Mpipi

Mpipi was designed specifically to overcome these limitations while retaining residue‐level resolution with an implicit solvent (Joseph et al. 2021). In principle, it is still a one‐bead‐per‐residue model, with the pair‐specific interaction matrix derived from atomistic potentials of mean force (PMFs) together with bioinformatics contact statistics. Importantly, single‐chain *R*_*g*_ values and experimental phase diagrams were used to validate the resulting model rather than as fitting targets, which is what makes the agreement reported below an out‐of‐sample result. In contrast to hydropathy‐based approaches, the ranking and magnitude of interactions explicitly encode the dominant role of π‐π and hybrid cation‐π/π‐π contacts, which lead Mpipi to achieve the near‐quantitative agreement with experimental *R*_*g*_ and *C*_sat_ for a diverse set of IDPs (Joseph et al. 2021). To further improve the electrostatics between non‐bonded residues, the generic Debye‐Hückel term is replaced by a pair‐specific, non‐symmetric Yukawa potential in a Mpipi‐Recharged variant, with parameters derived from atomistic PMFs for different pairs of charged residues and charged vs. neutral groups in explicit solvent and salt (Andrés et al. 2025). This means that the effective electrostatic attraction or repulsion between residues is not only a function of net charge and screening length, but also of the underlying chemistry and short‐range solvation structure, including charge asymmetries that are not included in a standard Debye‐Hückel description.

#### 4.1.4 CALVADOS

Complementary to Mpipi, the CALVADOS family provides another set of residue‐level, implicit‐solvent models that accurately link single‐chain statistics to phase behavior (Tesei et al. 2021). Building on the original CALVADOS formulation, CALVADOS2 refines the range of non‐ionic interactions and reoptimizes the residue‐specific parameters against a larger set of 55 proteins, thereby improving both the temperature scale and the transferability of the model across diverse sequences and salt conditions, while maintaining a Debye‐Hückel description of electrostatics (Tesei and Lindorff‐Larsen 2023). CALVADOS models are particularly useful for studying how temperature and salt modulate both single‐chain properties and condensate stability in a unified framework.

#### 4.1.5 1BPA

An early one‐bead‐per‐amino‐acid model, introduced by the Onck group (Ghavami et al. 2013, 2014) termed 1BPA, describes the inter‐ and intramolecular interactions between IDPs via a single *C*_α_‐centered bead, with bonded potentials derived from residue‐specific experimental Ramachandran statistics and non‐bonded terms that combine experimental residue‐specific hydrophobicities with Coulomb electrostatics (Ghavami et al. 2014). Originally developed for FG‐nucleoporins in the NPC and their interaction with folded nuclear transport receptors (de Vries et al. 2025; Dekker, de Vries, et al. 2025; Dekker et al. 2023; Fragasso et al. 2021; Ghavami et al. 2014; Jafarinia et al. 2024), the 1BPA model has recently been calibrated for RBPs and RBP‐RNA interactions to capture the conformational dynamics and phase separation of transcription factors and ALS‐related proteins and RNA (Driver and Onck 2025; Driver et al. 2024).

All these CG models indeed have quantitative and qualitative accuracy and can efficiently replicate the experimental outcomes under certain conditions. In general, the forcefields are developed for a particular region of sequence space, thermodynamic conditions, and target observables, and thus might not be equally accurate when applying to a diverse dataset.

### 4.2 Modeling Multidomain Proteins (MDPs)

Since many proteins contain both folded and unstructured domains, CG forcefields have been developed in recent years with the aim to account for both disordered and ordered regions (Latham and Zhang 2022), see Figure 3B. Traditional residue‐level models including HPS (Dignon et al. 2018), Mpipi (Joseph et al. 2021), and 1BPA (Driver and Onck 2025; Ghavami et al. 2014) are explicitly calibrated for IDPs, and might exhibit certain inaccuracies when applying to multidomain proteins (MDPs). Most of the biological systems, particularly viral assemblies, are composed almost entirely of MDPs, whose interdomain organization and linker flexibility control function and binding specificity (Carlson et al. 2022; Cubuk et al. 2021). Likewise, certain biomolecular condensates are formed by the interplay between folded proteins and RNAs. For example, the well‐characterized liquid–liquid nucleolar immiscibility into FC‐DFC‐GC phases are driven by core proteins, such as FBL and NPM1, which are MDPs and require a highly accurate model to computationally replicate the nucleolar morphology that is essential for rRNA processing (Feric et al. 2016; Mitrea et al. 2016).

Two philosophies underlie the current approaches. The first treats folded and disordered regions separately and holds the folded domains in their experimentally determined structures throughout the simulation, either as rigid bodies or through elastic network restraints (Section 4.2.1). These are the most widely used strategies because they are simple to parameterize and require no new interaction data, but they assume the native structure is rigid and does not deform, which potentially over‐stabilizes partially disordered segments within or adjacent to folded domains. The second one seeks a single transferable framework describing folded and disordered proteins on the same footing, so that structure is an emergent outcome rather than an input (Section 4.2.2).

#### 4.2.1 Preserving Native Structure: Rigid Bodies and ENM

A pragmatic strategy, often adopted in HPS‐based simulations, has been to treat the folded regions as rigid bodies, meaning that their native structures are kept intact by freezing all internal degrees of freedom, while disordered segments are modeled with a standard one‐bead‐per‐residue potential (Her et al. 2022). Although this strategy is computationally convenient and preserves the native fold, it suppresses internal rearrangements, partial unfolding, and allosteric transitions. These limitations become important when such conformational changes are directly coupled to binding or assembly, but they do not generally prevent meaningful predictions of phase behavior when the folded domains remain structurally intact. Indeed, related HPS‐type simulations have reproduced effects of the folded LAF‐1 domain on phase behavior (Dignon et al. 2018), phosphorylation‐ and DNA‐dependent HP1α condensation (Her et al. 2022), linker–SAM interactions regulating Polyhomeotic condensation (Gemeinhardt et al. 2025), and interdomain interaction networks in full‐length TDP‐43 (Mohanty et al. 2024). Thus, the suitability of rigid‐domain representations depends on whether internal folded‐domain dynamics are central to the biological question.

COCOMO2 introduces a surface‐exposure scaling factor that modulates the effective interaction strength of each residue based on its solvent accessibility, thereby weakening interactions for buried residues and preventing over‐sticky behavior of folded cores without changing the overall residue‐based parametrization (Jussupow et al. 2025). This modification accounts for considerably accurate interactions between folded‐domains, unfolded regions, and RNA within a unified coarse‐grained scheme.

CALVADOS3 further advances the multi‐domain modeling by shifting the bead representation from the *C*_α_ to the center‐of‐mass (COM) to remedy overly strong domain‐domain attractions caused by the *C*_α_‐centered representation and thus artificially compact multi‐domain conformations (Cao et al. 2024). Furthermore, the elastic network model (ENM) is applied based on the native structure, which means that pairs of residues within a certain threshold distance are connected via harmonic springs and the native folded structure remains preserved as a percolating network. This network of harmonic springs preserves the overall architecture and the residue‐specific hydrophobicities are optimized via a machine‐learning driven Bayesian optimization algorithm, based on the experimental *R*_*g*_ and *R*_*h*_ data of multidomain proteins (Cao et al. 2024). While the ENM can preserve the native secondary structure of a folded region, this approach is limited by the fact that the ENM‐stabilized domains can only fluctuate within a limited space that might be insufficient to capture large‐scale conformational dynamics, partial unfolding and disorder‐to‐order transitions at interfaces (Cao et al. 2024; Togashi and Flechsig 2018).

#### 4.2.2 Unified Description of Folded and Disordered Proteins

On the other hand, using a forcefield that is accurate only for folded proteins typically over‐stabilizes compact structures and underestimates chain expansion, whereas IDR‐optimized models tend to exaggerate flexibility and stickiness if used for globular domains, sometimes destabilizing native folds or producing unphysical domain‐domain aggregation (Best 2017; Robustelli et al. 2018). MOFF (Maximum‐Entropy Optimized Forcefield) is one of the first residue‐level CG models specifically designed to bridge this gap by combining maximum‐entropy optimization with least‐squares regressions (Latham and Zhang 2019, 2021). The model tunes an effective interaction matrix against a diverse set of experimental observables, primarily *R*_*g*_ and secondary‐structure data for IDPs, while simultaneously enforcing basic principles of energy‐landscape theory so that native structures of folded proteins are low‐energy minima separated from misfolded states. In principle, MOFF can reproduce the conformational statistics of IDPs and is capable of folding small globular proteins into their native structure (Latham and Zhang 2022). However, the model is mostly optimized for single‐chain properties and can misestimate quantitative phase behavior in crowded or highly charged systems, which limits its predictive power for complex multicomponent condensates.

Beyond single‐bead CG models, the 2BPA model has recently been introduced as an order‐to‐disorder framework aimed at capturing both disordered monomers and ordered aggregates within a unified description (Dekker, van der Klok, et al. 2025). Building upon 1BPA (Ghavami et al. 2013, 2014), the 2BPA model is extended by introducing an additional side‐chain bead, and two virtual hydrogen bond beads to account for β‐sheet formation and typical aggregation phenomena. The first implementation, 2BPA‐Q, was parameterized specifically for polyglutamine (polyQ) sequences by simultaneously fitting to all‐atom simulations and experimental data of disordered polyQ monomers and structured β‐sheet‐rich amyloid fibrils (Dekker, van der Klok, et al. 2025). The model has very recently been extended to all amino‐acids (termed 2BPA‐HB) in the context of disorder‐to‐order transitions in FG‐Nup condensates and amyloid fibers (Dekker et al. 2026) and the DNAJB6‐mediated suppression of polyQ‐aggregation (Adupa et al. 2026).

Another conceptually‐related development outside the forcefield domain is BioEmu, a generative deep‐learning system trained on static structures over 200‐ms of aggregated MD and experimental stabilities, which samples equilibrium ensembles including local unfolding and domain rearrangements at close to experimental free‐energy accuracy (Lewis et al. 2025). Although it is an ensemble generator rather than a forcefield, it still shares the ambition of a unified description and highlights the two obstacles: The first is the size and diversity of training data required for transferability while the second is the possibility that a fixed CG resolution may lack sufficient flexibility to reproduce an expanding range of observables. 2BPA is likely to meet this constraint as its scope is currently being extended from one kind of amino‐acid (Dekker, van der Klok, et al. 2025) to all amino‐acids (Adupa et al. 2026; Dekker et al. 2026). Overall, the balance between resolution, transferability, and efficiency for condensate‐scale RNA‐protein systems remains unresolved, but considerable progress is currently being made.

In general, the CG forcefields developed for MDPs represent substantial progress, but they still have certain limitations that are required to be addressed (Alberti et al. 2025).

### 4.3 RNA Modeling

The accurate modeling of RNA, on the other hand, is essential for a complete understanding of RNA‐protein interactions (Roden and Gladfelter 2021). Unlike proteins, where hydrophobicity scales and single‐bead representations have proven largely sufficient, RNA molecules introduce additional complexity through their polyelectrolyte character, sequence‐dependent base stacking and pairing interactions, and the critical role of divalent ions such as Mg^2+^ in modulating both structure and phase behavior (Nguyen et al. 2019; Tan and Chen 2011). A major challenge is that the same RNA chain can span multiple structural regimes, from disordered single strands and hairpins to tertiary folds, often within the same condensate (Roden and Gladfelter 2021). These electrostatics and structural features must be adequately captured by any CG model aiming to replicate the functional organization of RNA‐protein condensates. The fact that RNA is not merely a passive client but an active scaffold and modulator of condensate properties has driven substantial efforts to develop RNA‐compatible CG forcefields (Roden and Gladfelter 2021; Van Treeck and Parker 2018), see (Figure 3C and Table 1). This development follows two principal strategies, with one extending existing protein forcefields to include nucleotide parameters (Uusitalo et al. 2017), and the other developing dedicated RNA models that can be coupled with protein simulations through compatible interaction potentials (Driver et al. 2024).

#### 4.3.1 HPS‐Polynucleotide

Regy et al. for example, extended the HPS framework with a directly compatible one‐bead‐per‐nucleotide polynucleotide model (Regy et al. 2020). Each nucleotide is mapped onto a single bead carrying a unit negative charge, with short‐range interactions described by the same Ashbaugh‐Hatch form used for amino‐acids, so that protein and polynucleotide chains can be simulated together without additional cross‐terms. Applying the model to the disordered RGG domain of LAF‐1 mixed with RNA, the authors reproduced the experimentally observed modulation of protein phase separation by RNA, and examined how charge patterning and RNA incorporation govern the microscopic organization of the condensed phase (Regy et al. 2020). This established that a minimal one‐bead‐per‐nucleotide description is sufficient to capture heterotypic protein‐RNA phase behavior, and it remains the baseline against which higher‐resolution RNA models are assessed.

#### 4.3.2 CALVADOS‐RNA

CALVADOS‐RNA adopts a two‐beads‐per‐nucleotide representation based on a hydropathy‐like parametrization and is explicitly co‐parameterized with CALVADOS2 for IDRs (Yasuda et al. 2025). In this framework, bonded and stacking parameters were obtained bottom‐up by reproducing local RNA geometry from atomistic simulations, while the two stickiness parameters of the Ashbaugh‐Hatch potential (one for the backbone bead, one for the base bead) were fitted top‐down to SAXS‐derived *R*_*g*_ values of polyA_30_ and polyU_30_ across ionic strengths (Yasuda et al. 2025). This model is explicitly sequence‐independent, where a single parameter set is applied to all four nucleotides in a way that it does not resolve base‐specific recognition or secondary‐structure formation. It instead provides a quantitatively calibrated description of the generic polyelectrolyte and stacking behavior of disordered RNA that is directly compatible with CALVADOS2.

#### 4.3.3 Martini3‐RNA

Martini3‐RNA extends the Martini strategy of integrating top‐down and bottom‐up parameterization to nucleic acids, where bonded terms are refined against all‐atom RNA simulations while non‐bonded interactions are tuned by partitioning data and base–base potentials of mean force (Yangaliev and Ozkan 2025). This model maps nucleotides to a few beads (with explicit solvent) and includes an elastic network to preserve structural integrity, which enables accurate simulations for diverse RNA‐protein complexes. The trade‐off is that explicit‐solvent simulations are often heavy, meaning that large‐scale phase separation of RNA molecules can be computationally demanding (Kmiecik et al. 2016).

#### 4.3.4 3BPN‐1BPA

The 3BPN (three‐beads‐per‐nucleotide) model (Holmstrom et al. 2019) has been paired with 1BPA for simulating disordered RNA‐protein complexes enabling an efficient implicit solvent approach (Driver et al. 2024). In the 3BPN model, each nucleotide is represented by three beads (base, sugar, and phosphate), providing an explicit handling of chain stiffness and base stacking while remaining compatible with coarse protein forcefields.

#### 4.3.5 iConRNA

The above models, however, do not enforce explicit secondary structure beyond basic stacking, and treating base‐pairing in a generic way or not at all ultimately limits their scope to fully capture sequence‐dependent RNA recognition or complex folding topologies (Regy et al. 2020). Another prominent challenge in modeling RNA that has received insufficient attention is sequence specificity as most existing CG forcefields lack the ability to distinguish particular sequence motifs or capture their selective protein‐binding affinities (Iserman et al. 2020). Additionally, dynamic RNA folding and unfolding events are difficult to simulate because most CG models either assume RNA as a fixed secondary structure or treat it as fully flexible, missing the possibility of condition‐dependent (re)folding (Roden and Gladfelter 2021). This leaves unexplored how the crowded, multivalent environment inside condensates might alter RNA secondary structure or folding dynamics. To address this problem, a recent higher‐resolution model, iConRNA, has been developed, which assigns 6–7 beads for detailed base‐pairing, stacking and explicit Mg^2+^ ion exploration (Li and Chen 2025). This model correctly folds small RNAs and captures the effect of RNA sequence on phase behavior and the ensuing RNA conformational transitions. High computational demand is, indeed, a downside of iConRNA due to the large number of beads it assigns to the nucleotides.

Collectively, RNA modeling has emerged recently and substantial progress has been made to develop compatible forcefields that can accurately account for the role of RNA within RNA‐protein condensates (Regy et al. 2020; Roden and Gladfelter 2021), see Table 1 for an overview. However, the accurate treatment of RNA folding thermodynamics within a CG framework remains an active area of research, and current models may not fully capture the sequence‐specific effects that determine RNA structure and binding specificity.

## 5 Conclusion and Perspectives

The emerging integration of computational biophysics and structural biology has transformed our understanding of RNA‐protein interactions within biomolecular complexes, revealing that these assemblies are not merely passive repositories of macromolecules but rather dynamically regulated compartments with organized formation and function (Alberti et al. 2025; Mittag and Pappu 2022) (Figure 4). This study reviewed the molecular principles of three fundamental systems, that is, the nucleolus, nuclear speckles, and viral assemblies, where each represents distinct multivalent interactions between IDPs/MDPs and structured/unstructured RNA elements to maintain mesoscale organization (Carlson et al. 2022; Faraway et al. 2025; Lafontaine et al. 2021). Importantly, RNA itself can undergo phase transitions without proteins, which reveals crucial concepts of “RNA Physics” that may be shaped by counterion correlations (especially Mg^2+^), phosphate–phosphate bridging, and the formation of base‐stacking networks (Nguyen et al. 2019; Roden and Gladfelter 2021).

**FIGURE 4:**
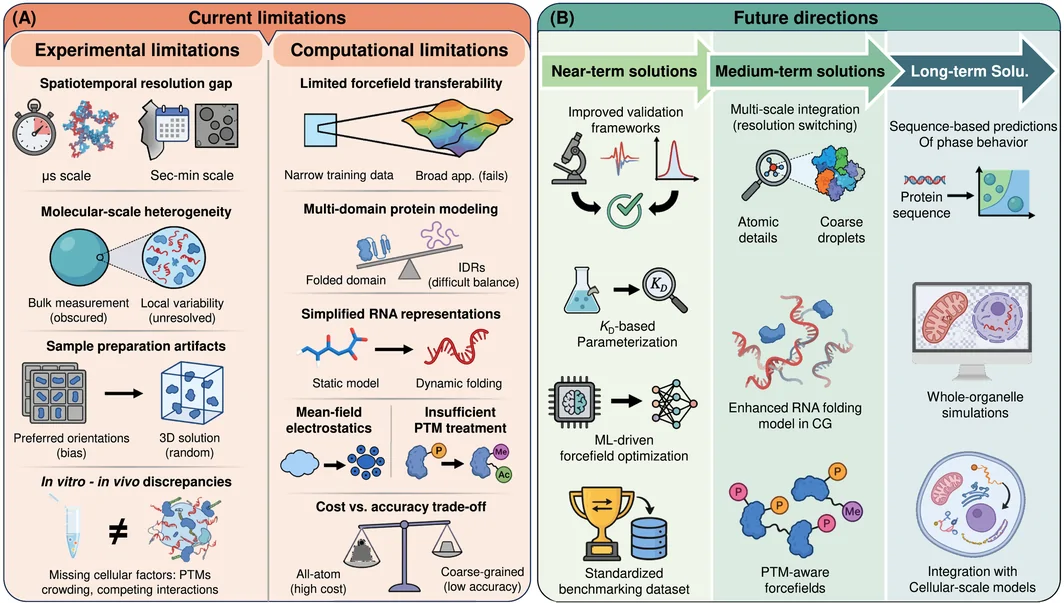
Current limitations and strategic roadmap for RNA‐protein complex modeling. (A) Overview of experimental (including spatiotemporal resolution gaps between imaging and simulation timescales, sample heterogeneity, and differences between reconstituted and cellular systems), and computational challenges (including limited forcefield transferability, difficulty in modeling MDPs, simplified RNA representations, and the cost‐accuracy trade‐off). (B) Overview of possible near‐term solutions (e.g., standardized benchmarks, *K*_*D*_‐based parameterization), medium‐term solutions (e.g., resolution‐switching simulations, improved RNA‐folding models, post‐translational modification (PTM)‐specific forcefields), and long‐term solutions (e.g., sequence‐based prediction of phase behavior, whole‐organelle simulations). PTMs are indicated schematically, including phosphorylation (P), acetylation (Ac), and methylation (Me).

In a multicomplex system, like the nucleolus, these principles can be well‐observed due to the well‐characterized molecular constituents and conserved tripartite organization (Feric et al. 2016; Lafontaine et al. 2021). The fact that FBL and NPM1 can form immiscible liquid phases (DFC‐GC) both in vivo and in vitro reconstituted systems, shows that the morphology arises from differential surface tension and sequence‐encoded interaction patterns (Feric et al. 2016; Mitrea et al. 2016). However, extending these principles from two‐component systems (FBL, NPM1) to the full complexity of the nucleolar proteome remains a challenge, due to the large variety of nucleolar proteins with diverse domain architecture and post‐translational modifications (PTMs) (Lafontaine et al. 2021; Quinodoz et al. 2025). On the other hand, current experimental approaches can measure bulk phase behavior and average material properties, but are limited by spatiotemporal resolutions to track the assembly pathways that drive mesoscale nucleolar organization (Alberti et al. 2025; Brangwynne et al. 2015). Here single‐molecule techniques are needed that can operate within crowded intracellular environments, supporting ensemble‐averaged measurements to capture the underlying high‐resolution heterogeneity and dynamics (Galbraith and Galbraith 2023). Likewise, super‐resolution microscopy methods (for example—simulated emission depletion) have potential to reveal nanoscale organization within condensates (He et al. 2023; Perego et al. 2023). However, correlating these structural observations with molecular composition and interaction networks might require the integration of imaging with mass spectrometry‐based proteomics and crosslinking approaches that remain technically demanding for MLOs (Markmiller et al. 2018; Youn et al. 2018).

When comparing the nucleolus to nuclear speckles, crucial similarities and distinctions can be noticed, as nuclear speckles exhibit liquid‐like properties (similar to the nucleolus) but feature fundamentally different assembly mechanisms and material organization (Faraway et al. 2025; Galganski et al. 2017). While the nucleolar architecture is scaffolded by nascent rRNA transcripts recruiting RBPs into hierarchical FC‐DFC‐GC, nuclear‐speckle formation depends critically on core scaffold proteins such as SON and SRRM2, whereas SR‐rich splicing factors and RNAs further regulate speckle composition, organization, and dynamics (Galganski et al. 2017; Ilik et al. 2020). This mechanistic distinction can be challenging to model as the forcefields optimized for RNA‐protein co‐condensation may not be accurate enough to capture the protein‐dominated NS assembly. Apart from this, the reported role of NS as organizational hubs for specific genomic loci reveals their functional importance beyond simple storage, a role that is poorly understood at the molecular level (Bhat et al. 2024; Ilık and Aktaş 2022). To understand the interplay between condensate‐mediated transcriptional regulation and chromatin organization, a multi‐scale approach accounting for condensate dynamics and gene expression programs may be required that is currently beyond the computational capabilities but can be an important goal for future research (Bhat et al. 2024; Mittag and Pappu 2022; Zhou et al. 2025).

Viral RNP assemblies have comparably distinct RNA‐protein interaction patterns, where sequence and architecture optimization for specific assembly pathways is managed by accurate genome packaging (Carlson et al. 2022; Iserman et al. 2020). This optimization can be effectively explained by the SARS‐CoV‐2 nucleocapsid protein that drives both LLPS for viral transcription and subsequent condensation into ribonucleosomes, packaging around 30 Kb genome into 40 particles/virion (Cubuk et al. 2021; Yao et al. 2020). While experimental characterization via cryo‐ET can directly compare simulated viral assemblies against experimental density maps at nanometer resolution, current CG forcefields are struggling to capture these disorder‐to‐order transitions (Klein, Cortese, et al. 2020). The reason might be the involvement of specific contact formation that is not encoded by isotropic, sequence‐averaged potentials that lack hydrogen bonding. In this regard, a 2BPA model may have potential to address this limitation as it explicitly incorporates hydrogen bonding interactions that stabilize β‐sheet formation during aggregation (Dekker, van der Klok, et al. 2025). Calibrating such a model to account for diverse structural transitions within viral assemblies can be challenging as it requires high‐resolution structural data for multiple viral systems. Furthermore, the physiological relevance of in vitro reconstitution studies remains uncertain, since cellular environments carry various host factors, PTMs, and competing interactions that may alter the apparent assembly kinetics (Ng et al. 2025).

The development of efficient CG forcefields has taken new directions with the emergence of models like HPS‐Urry (Regy et al. 2021), Mpipi (Joseph et al. 2021), CALVADOS (Tesei and Lindorff‐Larsen 2023), and 1BPA (Dekker et al. 2023; Driver and Onck 2025; Ghavami et al. 2014). These models have achieved quantitative agreement with the experimental measurements of single‐chain dimensions and phase boundaries. Furthermore, the accurate parametrization of hydrophobicity scales, and π‐π and cation‐π interactions as the driving force for many LLPS systems has demonstrated a convincing accuracy with close agreement with experimental data. Nevertheless, disagreements still arise regarding the relative strengths assigned to hydrophobic, π‐π, and cation‐π interactions, the treatment of electrostatics beyond the Debye‐Hückel approximation, and the balance between short‐range attractions and longer‐range repulsions that determines phase boundaries (Feito et al. 2025; Zeng et al. 2022). Based on the current CG approaches for biomolecular condensates there is a need for experimental transport properties (i.e., FRAP‐derived diffusion rates, viscosity etc.), interfacial tensions, and compositional partitioning as the ultimate benchmarking data, beyond standard experimental observables such as *R*_*g*_ and *R*_*h*_ (Alberti et al. 2025). Recent CG simulations have already begun to establish such connections, showing that sequence charge patterning jointly controls condensate diffusion, viscosity, surface tension, and intermolecular contact dynamics, and that these material properties correlate with dilute‐phase single‐chain conformations (Sundaravadivelu Devarajan et al. 2024). A close collaboration between experimental and computational groups may potentially enable such systematic validation, where experimental data made available at one end and compared with simulation predictions at the other. Crucially, this shortage of independent data is not only a validation problem but also bounds how sophisticated CG forcefields can usefully become. Following this, Increasing CG resolution introduces another trade‐off beyond computational cost that is greater structural or chemical detail generally requires more interaction parameters, which must be constrained by sufficiently diverse data. When the number of effective parameters grows faster than the available experimental or atomistic restraints, parameter identifiability decreases and overfitting becomes a concern, potentially reducing transferability beyond the training systems. Regularization and validation against independent and orthogonal observables are therefore essential when developing increasingly complex forcefields (Alberti et al. 2025; Liu et al. 2024; Tesei et al. 2021). Thus, increasing model resolution is beneficial only when accompanied by a corresponding increase in the quantity and diversity of data available for parameterization and validation. Apart from this, the development of new functionality in CG models is crucial. For example, extension of CG models to MDPs containing both folded and unstructured regions is critical (and challenging) as most phase‐separating proteins possess mixed architectures that both may have a role in driving condensation (Latham and Zhang 2022). Some strategies, including the combination of elastic network restraints for folded regions combined with standard IDP potentials for disordered regions are under discussion, and adopted by the CALVADOS3 (Cao et al. 2024) and COCOMO2 (Jussupow et al. 2025) models with the aim to preserve native structures while permitting realistic interdomain dynamics at the same time. The shift from *C*_α_‐centered to center‐of‐mass bead representations for folded domains, as implemented in CALVADOS3, addresses the systematic overestimation of folded domain‐domain attractions when IDR potentials are used for this (Cao et al. 2024). Nevertheless, these strategies assume the folded domains as completely or partially unchanged upon condensate formation, which might ignore the possibility of changes in protein conformation (Cao et al. 2024; Robustelli et al. 2018; Togashi and Flechsig 2018). For example, there is recent literature that report that condensate formation can both stabilize or destabilize folded domains (Hess and Joseph 2025; Tibble and Gross 2023). To overcome this limitation, developing forcefields might require the handling of crowded condensates potentially via solvent‐accessible surface‐area dependent (Dekker, van der Klok, et al. 2025) interaction parameters or multibody potentials.

PTM‐aware CG modeling has already begun to emerge. An early example is the extension of the 1BPA model to account for phosphorylation through modified charge and hydrophobicity. When applied to FG‐Nups, the model showed that phosphorylation expands the disordered chains, reduces FG‐network density, and alters NPC transport selectivity (Mishra et al. 2019). Perdikari et al. extended the HPS framework to explicitly represent phosphorylated Ser/Thr and acetylated Lys, demonstrating that both the number and positions of modifications can strongly alter IDP conformations and phase behavior (Perdikari et al. 2021). More recently, a CALVADOS‐compatible model parameterized against experimental dimensions was developed for phosphorylated Ser/Thr (Rauh et al. 2026), while PSFF‐PTM introduced PMF‐derived parameters for pSer, pThr, pTyr, acetyl‐Lys, and asymmetric dimethyl‐Arg that can be incorporated into CALVADOS and Mpipi (Mu and Lai 2026). Thus, the key future challenge is no longer simply to introduce PTMs, but to achieve broader and transferable coverage of different modification chemistries, combinations of multiple PTMs, and their effects on protein–protein and protein‐RNA interactions. Also, extending this capability to modified nucleotides will be particularly important for RNA‐protein condensates.

Another crucial development is to accurately represent RNA within CG simulations. Current RNA models, including 3BPN (Driver et al. 2024) and CALVADOS‐RNA (Yasuda et al. 2025), indeed account for essential features of polynucleotide chains (including charge density, backbone stiffness, and base‐pairing interactions) but assume initial RNA structures as disordered, potentially missing RNA (re)structuring in protein‐binding events. The recently developed iConRNA model employs high‐resolution representations that allow base‐pair formation during simulation, but at the expense of (substantially) higher computational cost (Li and Chen 2025). Other key extensions include PTM events, including 2′‐O‐methylation and pseudouridylation that produce diverse modified nucleotides (Sloan et al. 2017; Watkins and Bohnsack 2012). All in all, the development of accurate and efficient RNA‐compatible CG models should be considered as an important priority for the field.

The accurate parameterization of CG forcefields (both protein and RNA) require the availability of abundant experimental data that has become a major challenge (Best 2017; Mittag and Pappu 2022). Although *R*_*g*_ and *R*_*h*_ measurements provide critical benchmarks for single‐chain systems, the determination of phase boundaries requires system concentrations and solution conditions that are both challenging to control and may vary across different experimental techniques (Riback et al. 2017). The recent developments on reporting complete phase diagrams rather than only *C*_sat_ are indeed valuable, but the standardization of experimental protocols and data reporting formats are still challenging (Alberti et al. 2025; Arter et al. 2022; Saar et al. 2023). For protein‐RNA systems, the situation is even more challenging due to the heterogenous nature of the system (Regy et al. 2020). In this regard, techniques like fluorescence microscopy, and surface plasmon resonance might play an important role to systematically measure the protein‐RNA dissociation constants (*K*_*D*_) for forcefield optimization, but this is still largely unexplored (Ganser et al. 2022; Wang, Brasnett, et al. 2025).

The approach to use *K*_*D*_ as a benchmark can be further supported by the use of machine learning (ML), as these approaches have already shown remarkable results when utilized for forcefield optimization (Husic et al. 2020; Tesei et al. 2021). Benchmarking of CALVADOS‐3, for instance, relied on ML‐based Bayesian optimization framework to explore the high‐dimensional parametric space, with the purpose to reduce the root mean square deviation between experimental and simulated data for optimized residue‐specific interactions (Cao et al. 2024). These approaches can be promising to train and test ML‐models on *K*_*D*_ data for better forcefield calibration on multi‐domain proteins. The direct comparison between experimental and computational *K*_*D*_ can be supported by the use of available *K*_*D*_ calculation methods including the sub‐volume method, that are established validation frameworks (Fragasso et al. 2021; Jost Lopez et al. 2020; Kim and Hummer 2008). Progress on ML‐driven forcefields has been rapid and falls into three strands. The first retains a physics‐based functional form and uses ML only to optimize its parameters, as in the Bayesian learning of CALVADOS stickiness values (Cao et al. 2024; Tesei et al. 2021) and the maximum‐entropy optimization of the MOFF contact matrix (Latham and Zhang 2019). The second learns the potential itself bottom‐up, by force‐matching neural networks to all‐atom trajectories (i.e., from CGnet (Husic et al. 2020; Wang et al. 2019) to a transferable ML‐CG model that folds and unfolds proteins outside its training set (Charron et al. 2025)). The third learns directly from experimental observables by differentiating through the simulation trajectory, allowing neural‐network potentials to be trained top‐down on macroscopic data (Thaler and Zavadlav 2021). For condensates, however, the field remains almost entirely within the first strand, where transferable ML potentials have so far targeted globular protein folding rather than multi‐chain phase behavior. The availability of initial training data and the requirement for transferability across the vast sequence space of proteins and RNAs are major obstacles. These challenges can be minimized by incorporating physics‐based functional forms with ML‐optimized parameters for better accuracy and generalizability.

Considerable computational efforts have been made to address another challenge related to prediction of phase separation propensity from the amino acid sequences of IDRs (Wu, Wang, et al. 2025). Over 30 sequence‐based phase separation predictors have been reported thus far, with ~75% of them published since 2021 and ~30% released within 2024 alone (Wu, Wang, et al. 2025). In principle, these tools can be categorized into two mechanistic groups, where one involves physics‐based (CG‐mediated) (Ginell et al. 2025; Joseph et al. 2021; von Bülow et al. 2025) and other includes data driven (trained on experiments) (Hadarovich et al. 2024; Hatos et al. 2022; Hou et al. 2024; Liao et al. 2023) frameworks. Being first‐generation tools, PLAAC (prion‐like amino acid composition) (Lancaster et al. 2014), catGRANULE (disorder‐based, granule‐localisation propensity) (Bolognesi et al. 2016), and PScore (planar π‐contact frequency) (Vernon et al. 2018) were built on specific physicochemical features that might drive LLPS, but lack of consistent training/testing methodology limited their accuracy (Vernon and Forman‐Kay 2019). This limitation was addressed by second‐generation tools that incorporated broad training datasets into machine learning. DeePhase (Saar et al. 2021), for instance, uses sequence features to distinguish LLPS‐prone IDRs from structured proteins. Likewise, FuzDrop (Hatos et al. 2022) predicts phase separation propensity from entropy differences between bound and unbound states. PSAP (van Mierlo et al. 2021) and ParSe v2 (Wilson et al. 2023) are classifiers trained on curated in vitro and in vivo phase separation databases. Furthermore, successive predictors including PSPHunter (Sun, Qu, et al. 2024), Seq2Phase (Miyata and Iwasaki 2024), LLPhyScore (Cai et al. 2022), and MolPhase (Liang et al. 2024), further improved the differentiation between LLPS‐prone and non‐phase separation sequences. Performance evaluation via benchmarking on LLPSDB v2.0 showed that FuzDrop, DeePhase, and PSPredictor outperformed the first‐generation tools at correctly rejecting folded proteins as negatives, while LLPhyScore demonstrated highest overall performance (Liao et al. 2023). A recent study by Hou et al. (2024) presents a more accurate tool, PSPire, that integrates residue‐level and AlphaFold2 structure‐level features to improve identification of LLPS‐prone folded proteins, suggesting that structured proteins can also contribute to LLPS via multivalent interactions (Hou et al. 2024). Based on similar idea, PICNIC (Proteins Involved in CoNdensates in Cells) utilized both sequence and AlphaFold2 structural models to predict condensation regardless of structural disorder, and achieved 82% accuracy (highest thus far) (Hadarovich et al. 2024). Similarly, a recent update of catGRANULE 2.0 (Monti et al. 2025), further advances the prediction by also evaluating the effect of mutations on LLPS propensity. A recent study by von Bülow et al. (2025) reported a quantitative sequence‐based prediction framework that is built by combining CG simulations with active learning to predict LLPS free energies and *C*_sat_ directly from sequence (von Bülow et al. 2025). FINCHES (Forcefield‐based Interaction Network for Characterizing Heterotypic and Entropic Sequences), explores sequence‐driven chemical specificity between IDRs, resulting in direct prediction of phase diagrams, identification of interaction hotspots, and decomposition of chemically distinct IDR subdomains (Ginell et al. 2025). The aim of FINCHES is to employ CG‐derived mean‐field interaction parameters to estimate homo‐ and heterotypic interaction energies. While the homotypic protein–protein LLPS can now well‐predicted by opting above tools, the heterotypic interactions (resulted into LLPS) remain substantially harder to capture. For example, simulations and experimental results of prion‐like domains (PLDs) from FUS and hnRNPA1 demonstrate that complementary electrostatic interactions can drive co‐phase separation more readily than either component alone, where the condensate chemistry is shaped by the strength of homo‐ and heterotypic interactions (Farag et al. 2023). The FINCHES intermap provides a first approximation of IDR‐IDR chemical complementarity, but is explicitly restricted to qualitative or semi‐quantitative interpretation (Ginell et al. 2025). Recent work by Adachi and Kawaguchi (2024) links sequence charge patterning on IDRs to selective condensate partitioning of binding partners, which suggests that multivalent interactions among IDRs can produce specificity in condensate composition (without requiring ordered interface) (Adachi and Kawaguchi 2024). However, translating this phenomenon into predictive tools remain an open problem that gets even more challenging for IDR‐RNA interactions. Despite the crucial role of RNA in modulating phase behavior and condensate morphology, the efforts have been quite scarce. A recent attempt, RNAPSEC, showed that ML models (trained on protein and RNA sequence features) could achieve area‐under‐curve values of ~0.67, suggesting that the problem is tractable but with larger training dataset (Chin et al. 2024). The genuine gap is that there is currently no high throughput screening tool to predict RNA‐IDR phase separation from the joint sequence of an IDR and an RNA. CatGRANULE indirectly predicts RNA‐binding propensity via sequence‐level features of protein. Given the increasing interest in RNA‐protein condensates, the development of sequence‐driven IDR‐RNA LLPS predictors represent a clear computational need.

Finally, recent reports highlight connections between biomolecular assemblies and other biological barriers, particularly the NPC, through shared targeting principles such as nucleolar localization signals (NoLS) and nuclear localization and export signals (Detrés et al. 2025; Lu et al. 2021; Yang et al. 2023). Systematic comparison of the sequence features, interaction strengths, and phase behavior of nucleolar components and FG‐nucleoporins may explore fundamental principles that govern selectivity in disordered protein assemblies. The 1BPA forcefield, originally developed for FG‐Nups, may have the potential to accurately reveal the exact molecular mechanisms between these cellular components (Dekker et al. 2023; Driver and Onck 2025; Ghavami et al. 2014). Furthermore, the rapid developments in the understanding of RNA‐protein assemblies have opened new targets for advanced therapeutic strategies, given the important role of biological condensates in several diseases, including neurodegenerative diseases and cancer (Klein, Boija, et al. 2020; Uechi et al. 2025; Wang and Zhang 2024). Therefore, the development of transferable CG potentials for small chemical compounds that are compatible with the existing protein‐RNA models might potentially lead to new fundamental insights and open up a new route to pharmacological intervention.

## Author Contributions

**Muhammad Waleed Iqbal:** conceptualization, writing – original draft, visualization, investigation. **Patrick R. Onck:** writing – review and editing, supervision.

## Funding

This work was supported by the European Union's Horizon Europe research and innovation programme under grant agreement No. 101179819 (Evolve COFUND project).

## Conflicts of Interest

The authors declare no conflicts of interest.

## Data Availability

Data sharing not applicable to this article as no datasets were generated or analysed during the current study.
